# Chitosan-Based Thermogelling System for Nose-to-Brain Donepezil Delivery: Optimising Formulation Properties and Nasal Deposition Profile

**DOI:** 10.3390/pharmaceutics15061660

**Published:** 2023-06-05

**Authors:** Mirna Perkušić, Laura Nižić Nodilo, Ivo Ugrina, Drago Špoljarić, Cvijeta Jakobušić Brala, Ivan Pepić, Jasmina Lovrić, Maša Safundžić Kučuk, Marie Trenkel, Regina Scherließ, Dijana Zadravec, Livije Kalogjera, Anita Hafner

**Affiliations:** 1 Department of Pharmaceutical Technology, University of Zagreb Faculty of Pharmacy and Biochemistry, 10000 Zagreb, Croatia; mperkusic@pharma.hr (M.P.); lnizic@pharma.hr (L.N.N.); ipepic@pharma.hr (I.P.); jlovric@pharma.hr (J.L.); 2 Intellomics Ltd., 21000 Split, Croatia; ivo.ugrina@intellomics.com; 3 Visage Technologies d.o.o., 10000 Zagreb, Croatia; dspoljaric@gmail.com; 4 Department of Physical Chemistry, University of Zagreb Faculty of Pharmacy and Biochemistry, 10000 Zagreb, Croatia; cjakobus@pharma.hr; 5 Jadran-Galenski Laboratorij d.d., 51000 Rijeka, Croatia; masa.safundzic@jglpharma.com; 6 Department of Pharmaceutics and Biopharmaceutics, Faculty of Mathematics and Natural Sciences, Kiel University, 24118 Kiel, Germany; mtrenkel@pharmazie.uni-kiel.de (M.T.); rscherliess@pharmazie.uni-kiel.de (R.S.); 7 Priority Research Area Kiel Nano, Surface and Interface Sciences (KiNSIS), Kiel University, 24118 Kiel, Germany; 8 Department of Diagnostic and Interventional Radiology, University Hospital Center Sestre Milosrdnice, University of Zagreb School of Dental Medicine, 10000 Zagreb, Croatia; zadravec@sfzg.hr; 9 ORL/HNS Department, University Hospital Center Sestre Milosrdnice, Zagreb School of Medicine, 10000 Zagreb, Croatia; kalogjera@sfzg.hr

**Keywords:** donepezil, chitosan, nose-to-brain delivery, thermoresponsive in situ gelling system, 3D nasal cavity model, olfactory deposition

## Abstract

Donepezil nasal delivery strategies are being continuously investigated for advancing therapy in Alzheimer’s disease. The aim of this study was to develop a chitosan-based, donepezil-loaded thermogelling formulation tailored to meet all the requirements for efficient nose-to-brain delivery. A statistical design of the experiments was implemented for the optimisation of the formulation and/or administration parameters, with regard to formulation viscosity, gelling and spray properties, as well as its targeted nasal deposition within the 3D-printed nasal cavity model. The optimised formulation was further characterised in terms of stability, in vitro release, in vitro biocompatibility and permeability (using Calu-3 cells), ex vivo mucoadhesion (using porcine nasal mucosa), and in vivo irritability (using slug mucosal irritation assay). The applied research design resulted in the development of a sprayable donepezil delivery platform characterised by instant gelation at 34 °C and olfactory deposition reaching a remarkably high 71.8% of the applied dose. The optimised formulation showed prolonged drug release (*t*_1/2_ about 90 min), mucoadhesive behaviour, and reversible permeation enhancement, with a 20-fold increase in adhesion and a 1.5-fold increase in the apparent permeability coefficient in relation to the corresponding donepezil solution. The slug mucosal irritation assay demonstrated an acceptable irritability profile, indicating its potential for safe nasal delivery. It can be concluded that the developed thermogelling formulation showed great promise as an efficient donepezil brain-targeted delivery system. Furthermore, the formulation is worth investigating in vivo for final feasibility confirmation.

## 1. Introduction

Nasal drug delivery offers distinct advantages in brain-targeted therapy of neurologic disorders. Olfactory and trigeminal nerves innervating the olfactory and/or respiratory mucosa enable the direct transport of nasally administered drugs to the brain, bypassing the blood–brain barrier [1]. Nevertheless, the described potential is still scarcely utilised. The demands for a major step forward include the development of functional drug delivery systems and efficient drug deposition in specific nasal regions of interest [2]. Generally, research on the development of nasal delivery systems has addressed the issues of limited drug solubility, permeability, stability, and nasal residence time [3,4,5]. The potential of advanced delivery platforms of simple production and easy scale-up, such as in situ gelling liquid systems, is of particular interest [3,6]. These systems can incorporate mucoadhesive polymers, drug permeation enhancers, and/or nanocarriers, thus optimising nasal retention time, drug release, and absorption profile [7]. Formulation-related viscosity and/or reduced volume of administration may improve patient compliance, diminishing the potential for discomfort related to formulation run-off to the throat and unpleasant aftertaste [2,8].

Lately, drug delivery to the targeted region of the nasal cavity has become an inevitable milestone towards effective nasal therapy [4], and it emerged as one of the key elements in nasal product development [9]. Recently, our research team pioneered and extensively investigated the issue of coupling formulation and administration parameters employing the quality-by-design (QbD) approach to optimise the nasal deposition pattern in vitro in relation to the specific disease in adults [10]. Such an approach has been shown to hold great promise in promoting targeted nasal drug delivery [11,12].

Targeted nasal delivery can be considered a viable option for advancing drug therapy in Alzheimer’s disease [13]. Alzheimer’s disease is a progressive neurodegenerative disorder impairing memory, thinking, and behaviour, with rising prevalence corresponding to population ageing [14]. It is one of the leading indications of the demand for drug delivery strategies that overcome the blood–brain barrier [15]. Currently, donepezil is the first-line treatment in patients with mild to moderate Alzheimer’s disease, which is available predominantly in oral solid dosage forms [16]. Donepezil oral delivery has several limitations including first-pass metabolism, gastrointestinal and peripheral adverse effects, and low brain bioavailability [17]. On the contrary, the nasal administration of donepezil offers the potential for its efficient and direct central nervous system delivery, reduced systemic bioavailability, and a lower risk of adverse effects [18]. The recognised advantages of nasal donepezil delivery have led to the development of several liquid pharmaceutical platforms including donepezil nanosuspension [19], donepezil-loaded liposomes [20,21], nanoemulsion [22], lipid nanoparticles [23], microemulsion [24], and in situ gel [17], improving donepezil solubility and/or its permeation profile and enhancing its brain bioavailability, as evidenced in animal models in vivo. However, no studies on their possible application mode in humans and deposition pattern within the nasal cavity have been performed. 

The idea behind our study was to develop a donepezil-loaded advanced liquid formulation and harmonize its biopharmaceutical performance and nasal deposition profile while meeting all requirements for efficient brain-targeted delivery and keeping the formulating process simple. 

In situ gelling thermoresponsive polymer/drug solutions bear the potential to fulfil the above-mentioned requirements. They present easy-to-produce and scale-up formulations that can be readily applied as sprays while ensuring prolonged residence time in the nasal cavity upon gelation triggered by nasal physiological temperature [6]. Ideally, gel-forming constituents could contribute to a sustained drug release and interact with biological barriers to increase drug absorption, altogether resulting in increased bioavailability of the nasally applied drug [25].

Chitosan is a cationic linear polysaccharide widely applied as a mucoadhesive agent and absorption enhancer in nasal formulations [26]. Moreover, chitosan can yield thermoresponsive physical gels in the presence of β-glycerophosphate [27]. An almost neutral aqueous solution of chitosan and β-glycerophosphate demonstrates a strong rise in the storage modulus upon heating [6]. 

The aim of this study was to develop the chitosan-based donepezil-loaded thermoresponsive in situ gelling system, considering all aspects of donepezil nasal delivery, including formulation stability, sprayability, gelation temperature and time, drug release profile, interaction with the biological barrier, and, in particular, nasal deposition pattern, which was included as a required complementary indicator of the brain-targeting potential in humans and studied using the model of the healthy adult nasal cavity.

To the best of our best knowledge, this is the first report on the development of a chitosan-based thermoresponsive system for nasal delivery of donepezil. Previously, only Gu et al. [17] reported the development of a thermogelling nasal donepezil system. In that study, two types of poloxamers were used as the thermogelling agent. The systems were characterised in terms of gelation properties, in vitro release, and in vivo pharmacokinetics in rats, and formulation potential to increase donepezil brain bioavailability was confirmed. Compared with the aforementioned study, the innovation in our approach refers to a comprehensive research design that allows an integrated formulation development, taking into account all features of the nasal delivery, including the challenges of brain targeting in humans. 

The complex task of correlating the in situ gelling formulation properties and nasal deposition profile with formulation and/or nasal administration parameters was accomplished by employing a statistical design of experiments. 

Thorough biopharmaceutical characterisation of the leading formulation, including studies on stability, in vitro release, in vitro biocompatibility and permeability (using Calu-3 cells), ex vivo mucoadhesion (using excised nasal mucosa), and in vivo irritability (using slug mucosal irritation assay), was performed to evaluate the overall potential of the developed formulation in donepezil nose-to-brain delivery.

Finally, the results on the nasal deposition of the leading in situ gelling formulation will be compared with the results obtained previously with optimised donepezil powder formulation [12] to account for the differences in accessibility of the olfactory region using two distinct formulation approaches.

## 2. Materials and Methods

### 2.1. Materials

Donepezil hydrochloride (further denoted as DH) was obtained from Carbosynth Ltd. (Compton, UK). β-glycerophosphoric acid disodium salt pentahydrate (denoted as β-glycerophosphate, BGP, further in the text) was purchased from Biosynth Ltd. (Bratislava, Slovakia). Low-molecular-weight chitosan (molecular weight 50–190 kDa, with 75–85% degree of deacetylation; further denoted as chitosan, C), medium-molecular-weight chitosan (molecular weight 190–310 kDa, with 75–85% degree of deacetylation), and high-molecular-weight chitosan (molecular weight 310–375 kDa, with >75% degree of deacetylation) were obtained from Sigma-Aldrich (Taufkirchen, Germany). Simulated nasal fluid (SNF) was prepared as an aqueous solution by dissolving the following solids: NaCl (150.0 mM; Kemig, Zagreb, Croatia), KCl (40.0 mM; Kemig, Zagreb, Croatia), and CaCl_2_ × H_2_O (5.3 mM; Sigma-Aldrich, Taufkirchen, Germany) [10]. Hank’s balanced salt solution (HBSS; pH = 7.0) was prepared by dissolving the following in distilled water: KCl (5.4 mM), NaHCO_3_ (4.2 mM), NaCl (136.9 mM), and D-glucose monohydrate (5.6 mM), all purchased from Kemig, Zagreb, Croatia; KH_2_PO_4_ (0.4 mM; Kemika, Zagreb, Croatia); Na_2_HPO_4_ × 2H_2_O (0.3 mM; Fluka Chemie AG, Buchs, Switzerland); N-2-hydroxyethylpiperazine-N’-2-ethanesulfonic acid (HEPES; 30.0 mM; Sigma-Aldrich, Taufkirchen, Germany); and CaCl_2_ × 2H_2_O (1.3 mM; Sigma-Aldrich, Taufkirchen, Germany). The mixture was used for in vitro cell biocompatibility and permeability studies. Sar-gel^®^ (Arkema, Colombes, France) was used for the determination of the fractional deposition pattern. All other chemicals or solvents used in the study were of analytical grade and purchased from Kemika (Zagreb, Croatia).

### 2.2. Preliminary Studies

Thorough preliminary studies were performed to select suitable excipients and appropriate settings of upper and lower limits of the in situ gelling formulation and administration parameters. 

Preliminary samples were prepared according to the protocol described in Section 2.4. using chitosans of different molecular weight and different constituent concentrations as presented in the Supplementary Materials, Tables S2–S6.

Exclusion criteria included observed precipitation, inappropriate gelation time or temperature, and poor sprayability (determined as spray cone angle or droplet size distribution). Finally, administration parameters most common in the relevant literature were preliminarily tested within the design space of formulation parameters. The methods applied are described in Section 2.6.2, Section 2.6.3, Section 2.7, and Section 2.8.

### 2.3. Design of Experiments (DoE)

Quality by design (QbD) principles were built into the donepezil–chitosan–β-glycerophosphate (DH-C-BGP) in situ gelling system development process. This approach was used for optimising formulation and administration parameters to achieve efficient DH nasal delivery. Two formulation (the concentration of DH and C) and two administration (the inspiratory flow rate and angle of administration from the horizontal plane) parameters were incorporated in the definite screening design developed with JMP 14.0 statistical software (JMP^®^, Version 14.0, SAS Institute Inc., Cary, NC, USA, 1989–2007). Settings of the parameters in the experimental design are listed in Table 1.

Gelation properties (i.e., gelation time and temperature), zero-shear viscosity, droplet size distribution, spray cone angle, and deposition in the turbinate and olfactory region of the nasal cavity were investigated as responses. The statistical software JMP 14.0 (JMP^®^, Version 14.0, SAS Institute Inc., Cary, NC, USA, 1989–2007) was used to perform data analysis.

### 2.4. Preparation of the DH-C-BGP In Situ Gelling Formulation

DH-C-BGP nasal formulations were prepared according to Gholizadeh et al. [28], with slight modifications. First, concentrated C solutions (1.0%, 1.5%, and 2.0%, *w*/*w*) were prepared by dissolving chitosan in 0.5% (*v*/*v*) acetic acid at room temperature, under stirring conditions for 24 h. Then, an appropriate amount of DH was dissolved in a C solution. A concentrated BGP solution (49%, *w*/*w*) was prepared by dissolving BGP in water at room temperature. At 4 °C (in an ice bath), the concentrated BGP solution was added dropwise to the DH-C solution, followed by 10 min of mixing. DH-C and BGP solutions were mixed in a 1.6:1 volume ratio. Final DH and C concentrations are listed in Table 2. The concentration of BGP in all DoE samples was 188 mg mL^−1^. The pH value of all samples was also measured, using a S47 SevenMulti pH meter (Mettler Toledo, Greifensee, Switzerland).

### 2.5. Determination of DH Concentration in the DH-C-BGP Formulations

The DH concentrations in the prepared DoE samples were assessed via the high-performance liquid chromatography (HPLC) method, as detailed in Section 2.10. An aliquot of the DH-C-BGP sample was diluted with purified water. The diluted sample was filtered (0.2 µm pore size, Chromafil^®^ Xtra PES-20/25, Macherey-Nagel GmbH & Co. KG, Düren, Germany), and then the drug concentration in the sample was determined. Measurements for each sample were performed in triplicate.

### 2.6. Rheological Characterisation

Rheological characteristics of the prepared DH-C-BGP formulations were assessed using a Modular Compact Rheometer MCR 102 (Anton Paar GmbH, Graz, Austria). The temperature on the rheometer was assured via a Peltier temperature control system. Oscillatory rheological tests were performed using a parallel plate (PP50; diameter 50 mm) and rotational rheological tests were performed using a cone plate (CP50; diameter 50 mm, cone slope 1°). Software RheoCompass^TM^ Light Version 1.23.403 (Anton Paar GmbH, Graz, Austria) was used for the data analysis.

#### 2.6.1. Zero-Shear Viscosity Determination

A rotational creep test, using a CP50 measuring system, was applied to determine zero-shear viscosity (*η*_0_) of the prepared formulations, at 25 °C. The zero gap was set at 0.102 mm. Before the test, the sample was left on the lower plate to equilibrate at 25 °C for 3 min. During the test time of 5 min, a shear strain stress of 0.1 Pa was applied to the sample, and the shear strain was recorded as a function of time. Zero-shear viscosity was computed using RheoCompass software, fitting the shear stress vs. data on the shear rate and calculating the regression on the creep measuring data within the three retardation points [10]. Each sample was analysed in triplicate.

#### 2.6.2. Measurement of Gelation Temperature

In order to determine the sol–gel transition point, an oscillatory temperature test was used. A PP50 measuring system was used and the test recorded the changes in storage (*G*′) and loss modulus (*G*″) over a temperature range from 20 °C to 40 °C. The gap was set at 0.500 mm, the angular frequency was fixed at 6.28 rad s^−1^, and the applied strain was 1%. The intersection of *G*′ and *G*″ curves was recorded as the gelation temperature—*T*_GEL_ [29]. All measurements were performed in triplicate.

#### 2.6.3. Measurement of Gelation Time

The time needed for the formulation to undergo sol–gel transition was determined using an oscillatory time test. A PP50 measuring system was used and the test was performed at 34 °C—the temperature of the nasal cavity [5]. Variations in *G*′ and *G*″ were monitored as a function of time. The zero gap was fixed at 0.500 mm. The measurement was performed at an angular frequency of 6.28 rad s^−1^ and 1% strain. The cross point of *G*′ and *G*″ curves was recorded as the time needed for the formulation to transit from the liquid to the gel state (*t*_GEL_) [30]. Each formulation was analysed in triplicate.

### 2.7. Droplet Size Distribution (DSD)

Droplet size distribution (DSD) was determined via a Malvern Spraytec unit (Malvern Instruments, Malvern, Worcestershire, UK) via a laser diffraction technique. Samples were loaded into a VP7 spray pump equipped with a 232 NE actuator (dosing volume of 100 µL), kindly provided by AptarGroup Inc., Le Neubourg, France. Prior to the test, the spray pump was primed several times by discarding the actuations as waste. For the measurement, the tip of the device was placed 3 cm below the laser diffraction measurement zone. The focal distance from the lens was 300 mm. Each test was performed manually, in triplicate. The results were expressed as volume diameters *D*_v_10, *D*_v_50, and *D*_v_90. Span, defined as (*D*_v_90 − *D*_v_10)/*D*_v_50, was also calculated [31]. The analysis of the results was performed via Malvern Spraytec 3.20 software (Malvern Instruments, Malvern, Worcestershire, UK).

### 2.8. Spray Cone Angle Determination (SCA)

Spray cone angle (SCA) was determined by spraying the samples from the VP7 spray pump equipped with the 232 NE actuator (AptarGroup Inc., Le Neubourg, France) against the dark background. A Panasonic Lumix DMC-FZ1000 camera was used to record the spray plume under a set-up of 120 frames per second. The recorded spray plume was analysed using a virtual protractor. Each formulation was analysed in triplicate.

### 2.9. Assessment of the Deposition Pattern in the Nasal Cavity In Vitro

Nasal deposition pattern studies were performed using a multi-sectional 3D-printed nasal cast. The development of the nasal cavity model had been previously performed by our research group using anonymised Multislice Computer Tomography (CT) scan data of a 62-year-old patient [32], obtained from the Sestre milosrdnice University Hospital Center database. The protocol was carried out following the rules of the Declaration of Helsinki and was approved by the Ethics Committee of the Sestre milosrdnice University Hospital Center (Project identification code: EP-9941/19–3) and the Ethics Committee of the University of Zagreb Faculty of Pharmacy and Biochemistry (Class: 643–02/19–01/02; Registry number: 251–62-03–19-43). The model was made via stereolithography process using 3D Systems^®^ProX 800 (3D Systems, Inc., Rock Hill, SC, USA). Transparent rigid plastic Accura ClearVue was used to print the septum and the olfactory, respiratory, and posterior regions of the model. The anterior region was printed in a flexible material (Digital-Material FLX 9850, 60 ShoreA TangoBlackPlus and VeroWhitePlus) using a Stratasys Connex 350 printer (Stratasys Ltd., Rehovot, Israel) [32]. In order to observe a fractional deposition pattern, the model was divided into different regions of the nasal cavity: anterior region, turbinate region and septum—both with detachable olfactory fragments, and a posterior region (nasopharynx) with a connecting part for the respiratory pump. The model also contains the paranasal sinuses. The proper assembly and alignment of the model parts were ensured using bar pins, measuring 6.4 and 2.0 mm in diameter and 6.0 and 4.0 mm in height, and transverse coupling. 

The fractional nasal deposition pattern was assessed by placing the 3D-printed nasal cast on a stand in order to properly connect the model to a respiratory pump (model 613; Hardvard Apparatus, Holliston, MA, USA). The respiratory pump was used to simulate breathing conditions in a range from 15 L min^−1^ (rest breathing) to 30 L min^−1^ (deep moderate breathing condition) [10]. The inspiration flow rate set by the respiratory pump was checked via the inspiratory flow meter (In-Check Nasal; Clement Clarke International Ltd., Harlow, UK). Prior to the administration of the sample, the model was uniformly coated with a thin layer of a Sar-gel^®^ indicator paste (Arkema, Colombes, France) in order to visualise the deposition pattern and prevent the formulation from dripping. The Sar-gel^®^ paste turns purple when in contact with the formulation. The formulation was administered into the model using a VP7 spray pump, equipped with the 232 NE actuator. The spray pump was inserted into the right nostril, at a depth of 5 mm, and the device was actuated at an angle 0° from the vertical plane, and 45°, 60°, and 75° from the horizontal plane, while the left nostril was blocked. The fractional spray deposition pattern was determined gravimetrically: each region of the right side of the model was weighed before and after formulation administration, using an electronic balance (precision 0.01 mg; Mettler Toledo, Greifensee, Switzerland) [11]. The assessment of the deposition pattern was performed in duplicate for each run of the experimental design.

### 2.10. HPLC Method for Quantitative Determination of DH

The quantitative determination of DH was performed via the HPLC method using a 1260 Infinity II LC System consisting of an auto-sampling system, controller unit, degasser, UV–VIS detector and column oven (Agilent Technologies, Santa Clara, CA, USA). OpenLab software (Agilent Technologies, Santa Clara, CA, USA) was used to process the data of all chromatographic analyses. The chromatographic separation was carried out using a Kinetex C18 (250 × 4.6 mm, 2.6 µm particle size) reverse-phase column with a suitable guard column, both obtained by Phenomenex (Torrance, CA, USA). The method was performed according to Pappa et al. [33], with a few minor adjustments. Briefly, the mobile phase was prepared by mixing 0.02 M buffer phosphate (pH 2.7), methanol, and triethylamine in a volume ratio of 50:50:0.5. The flow rate was set at 1.0 mL min^−1^ and the injection volume at 20 µL. The analysis was performed at 25 °C. The detection wavelength was 268 nm. The run time was 7 min and the DH retention time was 5 min. The above-described method was validated based on the International Conference on Harmonization (ICH) guideline Q2 (R1) [34]. The validation of the method was carried out for linearity, range of linearity, accuracy, repeatability, intermediate precision, the limit of detection (LOD), and the limit of quantification (LOQ) (Table S1). All measured concentration values were within the range of linearity of the method (Table S1).

### 2.11. In-Depth Characterisation of the Leading DH-C-BGP Formulation

The leading DH-C-BGP formulation (LF) was further characterised in terms of in vitro DH release profile, mucoadhesiveness, biocompatibility, and permeability. The stability profile was also assessed. The slug mucosal irritation assay was performed in order to predict the formulation’s potential to cause nasal discomfort.

#### 2.11.1. In Vitro Release Studies

The automated Franz diffusion cell testing system Phoenix^TM^ RDS (Teledyne Hanson, Chatsworth, CA, USA) was used to determine the DH in vitro release profile from the LF. The Phoenix RDS platform consists of six vertical diffusion cells (volume of the receptor compartment: 15 mL) placed in the Peltier heating and stirring block. The cells were filled with SNF and the system was thermostated at 34 °C under a constant stirring of 500 rpm. A polyamide membrane with a pore size of 0.45 µm (Sartorius Stedim Biotech GmbH, Göttingen, Germany) was placed between the donor and acceptor compartment. Prior to the experiment, the membranes were conditioned in SNF for 15 min. The selected samples (500 µL) were pipetted into the donor compartment using a Multipette^®^ E3 (Eppendorf, Hamburg, Germany) equipped with 1 mL ViscoTip^®^ (Eppendorf, Hamburg, Germany). At predetermined time intervals, aliquots of 200 µL were drawn from the receptor compartment and replaced with fresh SNF, also heated to 34 °C. The total time of the experiment was 5 h and, during that time, the sink conditions were assured. The DH content in the collected samples was determined via the HPLC method, as detailed in Section 2.10. Donor compartments were also tested for DH content. All release experiments were performed in triplicate.

#### 2.11.2. In Vitro Mucoadhesion Test

Mucoadhesive properties of the LF were tested using a porcine nasal mucosa, obtained from a local slaughterhouse. The nasal mucosa was isolated from porcine heads by splitting the heads in half by longitudinal incision, and then the mucosa was separated from the septum and conchae [35]. Prior to the experiments, the mucosa was kept at −20 °C. A texture analyser TA.XT Plus (Stable Micro Systems, Godalming, UK), equipped with a mucoadhesion rig, was used to test the mucoadhesiveness of the LF; this method was previously developed by our research group [11]. Prior to the experiment, the mucosa was soaked in SNF for approximately 30 s at 34 °C. Then, the mucosa was cut into a 10 mm diameter disk and adhered to the upper probe using cyanoacrylate glue. A selected sample (100 µL) was pipetted onto the lower platform (using Multipette^®^ E3 (Eppendorf, Hamburg, Germany)) and thermostated at 34 °C for 30 s. The experimental parameters used were: pre-test, test, and post-test speeds of 0.5 mm s^−1^, 0.1 mm s^−1^, and 0.1 mm s^−1^, respectively, with a contact time of 120 s and applied force of 0.1 N. Mucoadhesive properties were expressed as the maximum detachment force (*F*_max_) and the work of adhesion (*W*_adh_) [32]. The sample from the DoE with a chitosan concentration different from the leading concentration (control formulation—CF) and corresponding aqueous DH solution were used as controls. Filter paper soaked in SNF served as a negative control. Each sample was tested in triplicate.

#### 2.11.3. Cell Culture Conditions

In order to examine in vitro biocompatibility and permeability of the formulations, Calu-3 cell line (ATCC^®^ HTB-55TM, ATCC, Manassas, VA, USA) was used. The cells were cultured in Dulbecco’s modified Eagle’s medium (DMEM-F12) cell culture medium (Sigma Aldrich, Burlington, MA, USA) containing penicillin/streptomycin (1% *v*/*v*; Lonza, Basel, Switzerland) and fetal bovine serum (FBS; 1% *v*/*v*, Sigma Aldrich, Burlington, MA, USA). The cell cultures were maintained at 95% humidity and 37 °C in an atmosphere of 5% CO_2_ (Sanyo CO_2_ incubator, Osaka, Japan). The medium was changed every 2 days, and the cells were passaged when they reached 70–90% confluence, according to the ATCC recommended protocol. The detachment of the cells from the flasks was performed using a mixture of trypsin (0.25%) and EDTA (0.02%) solutions in phosphate-buffered saline (PBS; Lonza, Basel, Switzerland).

#### 2.11.4. In Vitro Biocompatibility Study

The Calu-3 cells were seeded into 96-well plates (Corning Costar, Corning, NY, USA) at a density of 4 × 10^4^ cells per well and allowed to reach confluence over 48 h. Biocompatibility was assessed using an MTT (3-[4,5-dimethylthiazol-2-yl]-2,5-diphenyl tetrazolium bromide, Sigma-Aldrich, Burlington, MA, USA) colorimetric test.

Before the experiment, the cell culture medium from the wells was withdrawn, and the cells were washed with HBSS/HEPES (pH = 7.0) and treated with the prepared samples. The leading formulation was mixed with HBSS/HEPES (pH = 7.0) in a volume ratio 1:1. The LF-HBSS/HEPES (pH = 7.0) mixture resulted in DH, C, and BGP concentrations of 0.15 mg mL^−1^, 4.62 mg mL^−1^, and 94.00 mg mL^−1^, respectively. The cells incubated in HBSS/HEPES (pH = 7.0) were used as a negative control. The DH solution in HBSS/HEPES (pH = 7.0) (DH concentration ranging between 0.05 and 0.25 mg mL^−1^) and the BGP solution in HBSS/HEPES (pH = 7.0) (94.00 mg mL^−1^) served as controls. The cells were incubated with the prepared samples for 2 h at 37 °C. Afterwards, the samples were removed from the wells, and the wells were rinsed twice with HBSS/HEPES (pH = 7.0), followed by adding 100 µL of prepared MTT reagent in each well. The MTT reagent was prepared by dissolving MTT in PBS (MTT concentration of 2.5 mg mL^−1^), and then the solution was further diluted with DMEM-F12 to a final MTT concentration of 0.5 mg mL^−1^. The cells were incubated with MTT reagent for 2 h at 37 °C. After the incubation, the reagent was removed. Isopropanol (100 µL per well) was added to lyse the cells and to dissolve the formazan crystals. The amount of formazan product was determined using a spectrophotometer 1420 Multilabel counter VICTOR3, Perkin Elmer, Waltham, MA, USA, at 570 nm. Cell viability was calculated using the following equation:

(1) $$\mathrm{Viability}\;\left(\%\right)=\frac{A_{sample}-A_{ipr}}{A_{c}-A_{ipr}}\times 100$$

where *A_sample_* is the absorbance of the formazan crystal solution formed in cells treated with tested samples, *A_ipr_* is the absorbance of pure isopropanol, and *A_control_* is the absorbance of a solution of formazan crystals formed in cells treated only with HBSS/HEPES (pH = 7.0).

#### 2.11.5. In Vitro Permeability through the Epithelial Model Barrier

The Calu-3 epithelial cells were seeded into polycarbonate 12-well Transwell^®^ inserts, with 0.4 µm mean pore size, and 1.12 cm^2^ surface area (Corning Costar Inc., Corning, NY, USA) at a density of 5.5 × 10^5^ cells per well in order to test in vitro permeability [32]. The volume of the cell culture medium in the apical and basolateral compartment was 0.5 mL and 1.5 mL, respectively. After an incubation period of 48 h, the medium from the apical compartment was aspirated and the cells were cultured at the air–liquid interface, with 800 µL of the culture medium in the basolateral compartment. The media in the basolateral compartment was replaced with a fresh medium every 48 h. The cells were grown for 14 days until a plateau in transepithelial electrical resistance (TEER) was reached (above 1000 Ω cm^2^). An epithelial volt/ohm meter EVOM with STX-2 chopstick electrode (WPI Inc., Sarasota, FL, USA) was used to measure the TEER of the cell monolayers. Permeability studies included the following samples: (i) LF mixed with HBSS/HEPES (pH = 7.0) in a volume ratio of 1:1; (ii) solution of DH in HBSS/HEPES (pH = 7.0) (DH concentration = 0.25 mg mL^−1^); (iii) DH solution in hyperosmotic HBSS/HEPES (pH = 7.0) (DH concentration = 0.15 mg mL^−1^); and (iv) HBSS/HEPES (pH = 7.0) as the negative control. Osmolality of the hyperosmotic HBSS/HEPES (pH = 7.0) was equal to the leading formulation–HBSS/HEPES (pH = 7.0) mixture. The hyperosmotic HBSS/HEPES (pH = 7.0) was prepared according to Soni et al. [36]. An amount of 12 g of NaCl was added to 1 L of the prepared HBSS/HEPES (pH = 7.0), resulting in 2% NaCl (*w*/*v*) in the final hypertonic HBSS/HEPES (pH = 7.0) solution. Osmolality measurements were performed with an OsmoTECH^®^ Single-Sample Micro-Osmometer (Advanced Instruments, Norwood, MA, USA).

Before the experiment, the cells were washed with HBSS/HEPES (pH = 7.0) and, afterwards, the HBSS/HEPES (pH = 7.0) was pipetted into apical and basolateral compartments and the plate was incubated at 37 °C, 5% CO_2_, for 20 min. After the incubation, TEER was measured at the beginning of the permeability experiment. The HBSS/HEPES (pH = 7.0) was removed from the wells and 500 µL of each sample was added to the apical compartment. Each sample was tested in triplicate. Over the period of 120 min, 500 µL of the sample from the basolateral compartment was taken every 20 min and replaced with fresh HBSS/HEPES (pH = 7.0). All samples collected during the experiment, including the samples from the donor compartment at the 120 min final point of the experiment, were analysed for DH content using the HPLC method described in Section 2.10. In order to check cells’ monolayer integrity, TEER values were recorded during and upon completion of the experiments. During the permeability test, cells were incubated at 37 °C and 50 rpm on a horizontal orbital shaker. The apparent permeability coefficient (*P*_app_) was calculated according to the following equation:

(2) $$P_{\mathrm{app}=}\frac{dQ}{dt}\times \frac{1}{AC_{0}}$$

where d*Q*/d*t* is the permeability rate, *A* is the surface area of the permeation barrier, and *C*_0_ is the initial concentration of DH in the apical compartment [27]. 

The attenuation factor was calculated as the ratio between the *P*_app_ value of the LF or the DH solution in the hyperosmolar HBSS/HEPES (pH = 7.0) and the *P*_app_ value of the DH solution in the HBSS/HEPES (pH = 7.0) [12].

#### 2.11.6. Slug Mucosal Irritation Assay

The slug mucosal irritation (SMI) assay was used to evaluate the potential of the leading formulation to cause irritation on the nasal mucosa. The SMI was performed according to Trenkel et Scherließ [37]. Briefly, slugs of the species *Arion lusitanicus* were collected by wild harvesting. Prior to the experiment, the specimens were kept under laboratory conditions. The SMI assay was performed only with slugs with a body weight between 3 and 6 g. Slugs were weighed (BW) at the beginning of the experiment. An aliquot (100 µL) of the tested sample was transferred into a Petri dish and the mass of the dish and the sample was recorded. Slugs were placed on the liquid formulation for a contact period (CP) of 15 min. After the time of the first contact period expired, the slugs were placed on 1.5 mL of PBS in another dish, for a resting time of 60 min. During the resting time, the dish with the liquid formulation and the mucus produced after the first CP was weighed and recorded. This procedure was repeated two more times, so the total number of CPs was three. Total mucus production (TM) after three CPs was calculated according to the equation:

(3) $$\mathrm{TM}\;\left(\%\right)=\Sigma \;M\frac{\left(Mucus\;per\;CP,\;g\right)}{BW,\;g}\times 100\%$$

The same procedure was applied for negative control (100 µL of PBS) and positive control (100 µL of 1% (*w*/*v*) benzalkonium chloride (BAC) solution). Experiments were performed in triplicate. For each replicate, a separate slug was used.

#### 2.11.7. Stability Studies

Stability studies of the LF were performed in time points of 30 and 90 days. The liquid was kept in an airtight container, at 5 ± 3 °C, which is the envisaged storage temperature for the product. The drug concentration, rheological properties, SCA, and in vitro drug release profile were measured and compared with the initial results, recorded right after the preparation of the leading sample. All experiments were performed in triplicate.

### 2.12. Statistical Analysis

JMP 14.0 software (JMP^®^, Version 14.0, SAS Institute Inc., Cary, NC, USA, 1989–2007) was used to perform statistical analysis related to DoE, with *p* < 0.05 set as the minimal level of significance. Mucoadhesive properties were analysed using GraphPad Prism (trial version, GraphPad Software, Inc., San Diego, CA, USA), using a one-way analysis of variance (ANOVA), followed by a Tukey’s post hoc test. The similarity factor (*f*_2_) was used to assert the similarity between in vitro release profiles [38]. The profiles were considered similar when the *f*_2_ factor was greater than 50 [39].

## 3. Results and Discussion

The aim of this research was to develop a thermosensitive in situ gelling platform for efficient nose-to-brain delivery of DH. Thermosensitive hydrogels based on C-BGP have been studied for various purposes [29,40,41,42], but there are only a few studies aimed at the development of a C-BGP in situ gelling system for nasal delivery [28,43]. In this work, we focused on the development of a nasal C-BGP formulation that is safe, biocompatible, non-irritable, and efficient regarding delivery of DH to the brain. 

A nasal in situ gelling DH formulation was developed with the aim to produce a platform that is easily administered as a spray but turns to gel at the temperature of the nasal mucosa, 33–35 °C [44]. The sol–gel transition results in prolonged residence time of the formulation at the mucosal surface, owing to reduced mucociliary clearance [45].

Chitosan (C) is a non-toxic, biocompatible, biodegradable, and bioadhesive polymer with penetration-enhancing properties [30]. On its own, chitosan does not exhibit thermosensitive properties. However, by mixing the chitosan with a polyol-phosphate, such as BGP, the C-BGP platform undergoes a sol–gel transition at the temperature of the nasal cavity. At room temperature, β-glycerophosphate interacts with the positively charged chitosan amine groups, keeping chitosan chains in the solution by forming a water-protective shield despite increasing the pH of the solution. The mechanism of thermoreversible sol–gel transition includes a loss of electrostatic repulsion, ionic crosslinking, hydrogen bonding, and hydrophobic interaction [6]. 

### 3.1. Selection of the Formulation and Administration Parameters for DH-Loaded In Situ Gelling Systems

In this study, the development of a DH-loaded in situ gelling formulation was performed employing a statistical design of experiments. Such an approach potentiates the development of a DH thermogelling delivery platform with built-in quality, maximizing the cost and time savings [46]. In order to set the appropriate design space, thorough preliminary studies were conducted to select the constituents and their concentrations, fulfilling the requirements for nasal delivery [5]. 

Donepezil concentration range in the preliminary studies (0.3–0.6 mg mL^−1^; Table S2) was in line with the relevant references concerning donepezil nose-to-brain delivery [19]. Taking into account the dosing volume of 100 µL, the indicated concentration range provides delivering the dose per actuation equal to 0.6–1.2% (or 0.3–0.6%) of 5 (or 10) mg oral daily dose. The aforementioned fraction of the donepezil daily oral dose is considered appropriate to achieve its therapeutic potential via direct nose-to-brain delivery [47]. 

The starting BGP concentration was set at 188 mg mL^−1^ (Table S2), which is within the range usually reported for such systems [28,29,48]. In previous studies, the molecular weight of chitosan was shown to have a significant impact on thermogelling properties of the chitosan–BGP systems [41]. More particularly, it was observed that the increase in chitosan molecular weight resulted in faster system gelation [28,49,50]. In this study, DH-loaded systems prepared with the medium and high molecular weight chitosans at the gelation-promoting concentration (6.15 mg mL^−1^) showed precipitation or poor sprayability (Tables S2 and S3), while at lower chitosan concentrations, the systems did not exhibit gelling properties. On the contrary, the chitosans of low molecular weight provided appropriate spray and gelation properties at the relevant range of DH concentrations (Table S3).

In the next step, a DH-loaded system (DH concentration 0.3 mg mL^−1^) prepared with the low-molecular-weight chitosan (C concentration 6.15 mg mL^−1^) was selected for the BGP concentration optimization. Based on the results obtained (Table S4), a fixed concentration of BGP was set. Namely, even a small shift in gelling agent concentration caused a great difference in the formulation’s thermogelling properties [51]. Ranges in the BGP concentrations were previously reported in experimental designs for the development of similar systems [52,53]. However, susceptibility to the BGP concentration in this study may be related to the difference in the incorporated drug type and concentration.

The final step revealed concentration ranges for both the DH and the low molecular weight chitosan, defining the design space for formulation optimisation in relation to the studied responses (Tables S5 and S6). 

It was previously shown that the angles of administration and inspiratory flow exhibit a pronounced impact on the nasal deposition pattern. According to the literature sources, administration parameters including the angle of administration from the horizontal plane ranging from 30 to 75° and inspiratory flow from 0 to 30 L min^−1^ were assessed in the preliminary deposition studies [10,54,55,56,57]. Only the angle of administration of 30° was discarded as inappropriate for targeted nasal delivery of tested formulations. Other parameters were included in the statistical design of experiments as presented in Table 1. 

### 3.2. Design of Experiments: Optimisation of DH-C-BGP Formulation

The design of the experiments was successfully incorporated into the DH-C-BGP formulation development. The DoE generated 17 runs varying in formulation (DH and C concentration) and/or administration parameters (horizontal angle of administration and inspiratory flow rate). The design matrix is presented in Table 2.

The DH concentration in the prepared DoE samples ranged between 0.29 ± 0.00 and 0.52 ± 0.02 mg mL^−1^ (Table 2), providing complete DH dissolution in C-BGP systems and adequate DH dose with respect to nasal delivery [19]. The value of pH for all prepared samples ranged between 7.02 ± 0.00 and 7.35 ± 0.00 (Table 2). The observed pH values were expected due to the basic values of BGP systems [28] and are acceptable for nasal administration [58,59].

Within the DoE, the formulation of rheological and thermogelling properties (gelation time and temperature, and zero-shear viscosity), spray characteristics (droplet size distribution and spray cone angle), as well as olfactory and turbinate deposition assessed in the nasal cavity model were analysed as responses. The obtained results are presented in Table 2.

Regression modelling was applied to enlighten which formulation parameters (and their interactions) had the most influence on the responses and to select the parameter settings that resulted in the most convenient response values. Regression modelling equations are presented in standardised covariates that are normalised to unitless intervals [−1, 1], an approach common in experimental design modelling [60]. Within the regression model equation, all statistically significant parameters (*p* < 0.05) are noted with an asterisk. The regression models and the analysis for the formulation of rheological, thermogelling, and spray properties as DoE responses are presented in Table 3. Nasal deposition modelling in relation to the formulation and administration parameters will be discussed in a separate section. Prediction profilers are presented in Figures S1–S7. 

#### 3.2.1. Formulation of Rheological, Thermogelling, and Spray Properties within the DoE Space

DH-C-BGP formulations prepared according to the design matrix were moderately viscous solutions with zero-shear viscosity ranging between 35.03 ± 0.82 and 232.21 ± 2.30 mPa s. The observed range of the viscosity values is appropriate for simple administration by spraying [2]. In addition, DH-C-BGP systems exhibit shear-thinning behaviour [28], which implies a decrease in viscosity at the applied aerosolisation shear stress. 

All DoE samples gelled at the physiological temperature of the nasal mucosa (34 °C) with the gelation time (*t*_GEL_) ranging between 0.0 ± 0.0 and 14.9 ± 0.2 min (Table 2), which is below the time of nasal mucus turnover (approximately 20 min) [61]. As expected, the increase in gelation time at 34 °C was coupled with the increase in the temperature of the instant gelation (*T*_GEL_), which ranged from 32.2 ± 0.9 to 39.9 ± 0.1 °C within the DoE space. Nonetheless, DoE settings revealed the potential for optimising this crucial formulation property to undergo instant gelation at the temperature of the nasal mucosa.

The spray cone angle (SCA) of the prepared DoE samples was between 15.1 ± 0.3 and 26.6 ± 1.2° (Table 2). The SCA is an important factor that affects nasal deposition and, therefore, the efficacy of a nasally administered drug. The narrow spray cone angles obtained in this study favour targeted nasal deposition [5,57]. The olfactory region of the nasal cavity is a small area (representing ~5–7% of the nasal epithelial surface area) located at the roof of the nasal cavity [62]. It is more likely that the nasal spray will reach the olfactory region if it is concentrated in a narrow plume. Apart from SCA, it is important to note that the deposition in the olfactory region is also affected by the droplet size distribution and the velocity of the applied spray [63].

For all DoE samples, values for *D*_v_10, *D*_v_50, and *D*_v_90 were as follows 23.6 ± 1.4–72.3 ± 3.2 µm, 61.6 ± 4.8–167.6 ± 6.3 µm, and 137.2 ± 7.3–320.6 ± 15.2 µm, respectively (Table 2). The measured DSD values are in accordance with regulatory requirements for nasal sprays (the vast majority of droplets being larger than 10 microns) [31,64]. The span ranged from 1.45 ± 0.06 to 1.89 ± 0.06 (Table 2). A span smaller than 2.0 indicates a monodisperse system with narrow size distribution [65]. Droplets with a narrow size distribution are more likely to deposit uniformly to the targeted area (e.g., olfactory region) in comparison with droplets with a broad size distribution. This could be explained by the fact that droplets with a narrow size distribution have more consistent aerodynamic behaviour and are less likely to undergo deposition by inertial impaction, which can cause uneven deposition in the nasal cavity [56,63,66].

**Table 2:** Sample sequence from the design of the experiment and the corresponding DC (drug concentration), pH, zero-shear viscosity (*η*_0_), gelation temperature (*T*_GEL_), gelation time (*t*_GEL_), spray cone angle (SCA), droplet size distribution (*D*_v_10, *D*_v_50, and *D*_v_90) and span, olfactory deposition (OD), and turbinate deposition (TD).

	*c*_DH_ (mg mL^−1^)	*c*_C_ (mg mL^−1^)	IFR * (L min^−1^)	AAH * (°)	DC (mg mL^−1^)	pH	*η*_0_ (mPa s)	*T*_GEL_ (°)	*t*_GEL_ (min)	SCA (°)	*D*_v_10 (µm)	*D*_v_50 (µm)	*D*_v_90 (µm)	Span	OD (%)	TD (%)
1	0.30	6.15	0	75	0.30 ± 0.00	7.35 ± 0.00	35.26 ± 1.11	34.2 ± 0.3	1.3 ± 0.7	26.6 ± 1.2	23.6 ± 1.4	61.6 ± 4.8	140.0 ± 7.1	1.9 ± 0.1	65.9 ± 1.6	11.4 ± 1.6
2	0.30	6.15	30	45	0.29 ± 0.00	7.28 ± 0.01	36.49 ± 0.03	33.8 ± 0.4	1.2 ± 0.6	24.9 ± 0.2	26.5 ± 1.3	71.1 ± 9.4	153.5 ± 17.8	1.8 ± 0.0	1.9 ± 1.0	62.7 ± 1.0
3	0.30	6.15	30	75	0.30 ± 0.00	7.24 ± 0.00	35.03 ± 0.82	34.9 ± 0.6	1.0 ± 0.4	25.5 ± 0.7	29.1 ± 2.8	87.0 ± 10.2	184.2 ± 14.9	1.8 ± 0.1	28.3 ± 1.6	60.3 ± 1.6
4	0.30	7.69	0	45	0.31 ± 0.02	7.12 ± 0.02	88.30 ± 1.81	34.2 ± 0.8	1.2 ± 1.0	19.6 ± 0.1	39.2 ± 6.3	120.5 ± 13.2	240.1 ± 19.7	1.7 ± 0.1	3.5 ± 3.3	38.3 ± 3.3
5	0.30	9.23	0	75	0.30 ± 0.00	7.02 ± 0.00	206.37 ± 1.46	33.7 ± 0.1	0.0 ± 0.0	15.5 ± 0.4	69.8 ± 3.7	161.1 ± 5.2	309.7 ± 10.9	1.5 ± 0.0	71.8 ± 0.8	10.5 ± 0.8
6	0.30	9.23	15	45	0.30 ± 0.00	7.06 ± 0.01	194.28 ± 5.27	32.2 ± 0.9	0.0 ± 0.0	16.4 ± 1.6	42.5 ± 4.1	115.2 ± 4.5	226.5 ± 7.1	1.6 ± 0.1	10.6 ± 0.1	63.8 ± 0.1
7	0.30	9.23	30	60	0.30 ± 0.00	7.03 ± 0.01	201.55 ± 0.46	33.4 ± 1.0	0.0 ± 0.0	15.6 ± 0.2	46.8 ± 1.4	129.5 ± 1.5	255.3 ± 1.7	1.6 ± 0.0	21.4 ± 0.3	40.3 ± 0.3
8	0.40	6.15	0	45	0.42 ± 0.02	7.15 ± 0.00	37.08 ± 0.24	36.9 ± 0.2	5.1 ± 0.4	23.1 ± 0.6	25.9 ± 2.3	72.8 ± 9.7	154.8 ± 17.0	1.8 ± 0.0	7.9 ± 3.3	65.1 ± 3.3
9	0.40	7.69	15	60	0.41 ± 0.01	7.09 ± 0.00	90.02 ± 0.38	36.3 ± 0.6	3.2 ± 0.3	19.6 ± 0.3	45.0 ± 8.9	129.3 ± 15.6	250.9 ± 25.9	1.6 ± 0.1	29.4 ± 3.9	49.2 ± 3.9
10	0.40	9.23	30	75	0.40 ± 0.00	7.06 ± 0.01	225.58 ± 3.33	33.7 ± 0.2	0.1 ± 0.2	15.5 ± 0.4	72.0 ± 3.3	166.2 ± 7.6	315.2 ± 17.9	1.5 ± 0.0	35.0 ± 3.4	41.4 ± 3.4
11	0.50	6.15	0	60	0.50 ± 0.00	7.11 ± 0.00	39.70 ± 1.45	39.9 ± 0.1	13.9 ± 0.9	23.3 ± 0.2	23.8 ± 0.9	61.7 ± 4.3	137.2 ± 7.3	1.8 ± 0.0	31.4 ± 5.0	31.7 ± 5.0
12	0.50	6.15	15	75	0.50 ± 0.00	7.21 ± 0.03	39.29 ± 0.59	38.0 ± 0.3	14.3 ± 1.2	23.2 ± 1.1	26.5 ± 1.1	66.3 ± 4.3	141.6 ± 7.3	1.7 ± 0.0	45.6 ± 1.3	26.6 ± 1.3
13	0.50	6.15	30	45	0.49 ± 0.00	7.15 ± 0.00	37.67 ± 0.55	39.2 ± 0.5	14.9 ± 0.2	23.0 ± 0.4	26.7 ± 1.0	68.4 ± 2.6	145.4 ± 4.2	1.7 ± 0.0	6.8 ± 0.3	31.5 ± 0.3
14	0.50	7.69	30	75	0.52 ± 0.02	7.07 ± 0.01	91.15 ± 0.87	38.5 ± 0.1	11.2 ± 0.0	18.7 ± 0.7	47.3 ± 7.3	133.4 ± 11.6	258.2 ± 16.7	1.6 ± 0.1	25.5 ± 5.7	32.9 ± 5.7
15	0.50	9.23	0	45	0.50 ± 0.01	7.03 ± 0.02	209.01 ± 2.98	35.3 ± 0.4	4.4 ± 1.0	16.1 ± 1.0	70.9 ± 14.0	162.3 ± 18.3	306.1 ± 31.0	1.5 ± 0.1	1.9 ± 2.1	60.2 ± 2.1
16	0.50	9.23	0	75	0.50 ± 0.01	7.04 ± 0.00	232.21 ± 2.30	35.7 ± 0.1	2.1 ± 0.4	15.1 ± 0.3	72.3 ± 3.2	167.6 ± 6.3	320.6 ± 15.2	1.5 ± 0.0	22.9 ± 4.7	42.5 ± 4.7
17	0.50	9.23	30	45	0.49 ± 0.00	7.06 ± 0.01	218.45 ± 2.33	35.5 ± 1.6	4.2 ± 0.3	15.6 ± 0.7	61.7 ± 3.8	150.1 ± 7.6	286.6 ± 15.3	1.5 ± 0.0	6.1 ± 1.2	63.9 ± 1.2

*c*_DH_ = donepezil hydrochloride concentration; *c*_C_ = chitosan concentration in the spray-drying solution; IFR = inspiratory flow rate; and AAH = administration angle in relation to horizontal plane. All samples were prepared at a BGP concentration of 188.00 mg mL^−1^. * Administration parameters related to the deposition studies. Values for the responses are mean ± SD, n = 3, except OD and TD where n = 2.

##### Regression Modelling

The statistical analysis of the regression models for zero-shear viscosity, gelation time and temperature, spray cone angle, and volume diameters or spray droplets revealed a good fit (Table 3). Each of the aforementioned responses was significantly influenced by DH and CH concentrations (apart or in interaction), confirming the rationale for their consideration as DoE variables. 

##### Impact of Chitosan Concentration

Chitosan concentration revealed linear and/or quadratic impacts on the zero-shear viscosity, gelation time, gelation temperature, spray cone angle, and droplet size distribution (Table 3). 

An increase in chitosan concentration led to increased zero-shear viscosity, owing to the entanglement of the polymer chains that led to the limited movement of individual chains and the rise in zero-shear viscosity [67]. At the same time, the increase in C concentration decreased gelation time and temperature, which is in accordance with the literature [41,68]. Namely, due to the entanglements of the polymeric chains, less heat is needed to create a 3D gel network. However, too high a concentration of chitosan can slow down the gelation process. Due to the high chitosan concentration, the solution becomes highly viscous, and the attraction between the chitosan amino groups and the BGP phosphate groups is hindered [51]. In conclusion, setting the right interval of chitosan concentration in the preliminary studies was crucial to obtain valid regression modelling for gelation properties. 

The increase in C concentration resulted in a spray cone angle decrease and spray droplet size increase, both of which are related to increased solution viscosity [55,66,69]. Namely, the increased viscosity of the nasal spray led to the decrease in the velocity of the spray droplets as they exited the nozzle. Thereupon, the droplets of the spray spread out less and formed a narrower spray cone angle [69,70]. The increased viscosity resulted in the production of larger droplets in the aerosolisation process [56]. Larger droplets tend to travel in a narrower direction and will ultimately be deposited in the narrower area into which they are directed [66]. 

##### Impact of DH Concentration

DH concentration exhibited a significant influence on the monitored rheological thermogelling and spray properties, as a single parameter and/or in combination with the C concentration (Table 3). The increase in DH concentration led to the increase in zero-shear viscosity. This effect can be explained by the presence of DH molecules in the chitosan network that increased the steric repulsion between the polymer chains, increasing the system viscosity [71]. The same reason could be behind the decrease in the spray cone angle that occurred with the increase in DH concentration.

The rising DH concentration in the C-BGP thermosensitive platform increased the gelation time and temperature. As explained above, the addition of the drug in the system increased the viscosity, and the diffusion of the heat was reduced [71]. Moreover, the addition of salt decreased the pKa of the BGP and consequently led to the chitosan’s higher degree of ionisation; hence, more heat was needed for gel formation [72]. 

The interaction between the DH and C concentrations within the derived regression model for the gelation time (Table 3) could be explained by the presumption that the DH affected the steric repulsions between the polymer chains to an extent that was dependent on the C concentration in the system [72,73,74].

**Table 3:** The results of the statistical analysis on zero-shear viscosity, gelation time, gelation temperature, spray cone angle, and droplet size distribution within the DoE.

Common DoE Response	Regression Model	Regression Analysis			
		R^2^	RMSE	PRESS R^2^	PRESS RMSE
Zero-shear viscosity	*η*_0_ = 93.50 * + 5.01 × *c*_DH_ * + 87.63 × *c*_C_ * + 3.97 × *c*_DH_ × *c*_C_ − 5.52 × *c*_DH_^2^ * + 36.08 × *c*_C_^2^ *	1.00	6.79	0.99	8.53
Gelation time	*t*_GEL_ = 3.60 * + 4.31 × *c*_DH_ * − 2.92 × *c*_C_ * − 2.41 × *c*_DH_ × *c*_C_ * + 2.41 × *c*_DH_^2^ * − 1.19 × *c*_C_^2^ *	0.99	0.72	0.97	0.91
Gelation temperature	*T*_GEL_ (°) = 35.61 * + 1.84 × *c*_DH_ * − 1.24 × *c*_C_ *	0.87	0.86	0.80	0.96
Spray cone angle	SCA(°) = 19.02 * − 0.65 × *c*_DH_ * − 4.27 × *c*_C_ * + 0.57 × *c*_DH_ × *c*_C_ * + 0.43 × *c*_DH_^2^ + 0.58 × *c*_C_^2^	0.99	0.59	0.96	0.76
Droplet size distribution	*D*_v_10 (µm) = 43.82 * + 3.69 × *c*_DH_ + 18.14 × *c*_C_ * + 4.00 × *c*_DH_ × *c*_C_ + 0.32 × *c*_C_^2^	0.89	7.25	0.78	8.45
	*D*_v_50 (µm) = 127.74 * + 4.56 × *c*_DH_ + 40.22 × *c*_C_ * + 8.12 × *c*_DH_ × *c*_C_ * − 17.69 × *c*_C_^2^ *	0.93	12.70	0.86	14.87
	*D*_v_90 (µm) = 249.71 * − 6.17 × *c*_DH_ + 68.80 × *c*_C_ * + 14.60 × *c*_DH_ × *c*_C_ * − 29.94 × *c*_C_^2^	0.92	22.51	0.84	26.41

*c*_DH_ = donepezil hydrochloride concentration; *c*_C_ = chitosan concentration in the spray-drying solution. R^2^ = the coefficient of determination; RMSE = root mean square error; and PRESS = predicted residual error sum of squares. * Statistically significant parameters (individual and in interaction; *p* < 0.05).

#### 3.2.2. Nasal Deposition of DH-C-BGP Formulations

A growing number of studies that are tackling nose-to-brain delivery of drugs have emerged over the past decade [2]. However, the question of how to deliver drugs to the small, hindered area of the olfactory zone still remains a major obstacle. For neurological drugs to manifest their optimal therapeutic effect, the goal is to achieve high drug concentrations in the previously mentioned area. Apart from targeting the olfactory region, a direct delivery to the brain is enabled through the trigeminal nerve innervating both the olfactory and respiratory mucosa [75,76,77]. 

To ensure the targeted delivery of the nasal drug, deposition studies should be implemented in the early phase of formulation development [2]. In this work, nasal deposition studies were performed using a multi-sectional 3D-printed model based on the CT scan of a patient with healthy airway passages. Inflammatory disorders, such as rhinitis or rhinosinusitis, may influence the nasal deposition pattern [9,78]. However, they are not common for patients with Alzheimer’s disease [79]. Hence, a healthy phenotype of the nasal cavity is chosen for this study. The smallest vertical cross-sectional areas (valve region) and the length of the nasal cavity fit into the ‘normative range’ [32,80]. The model was connected to the respiratory pump to simulate three breathing patterns: no breathing, rest breathing, and deep moderate breathing [81,82,83]. One nostril was closed during the actuation [55]. For the nasal device, we used an Aptar’s VP7 pump, which was previously also used in other studies [56,84].

##### Deposition in the Olfactory Region

**Figure 1:**
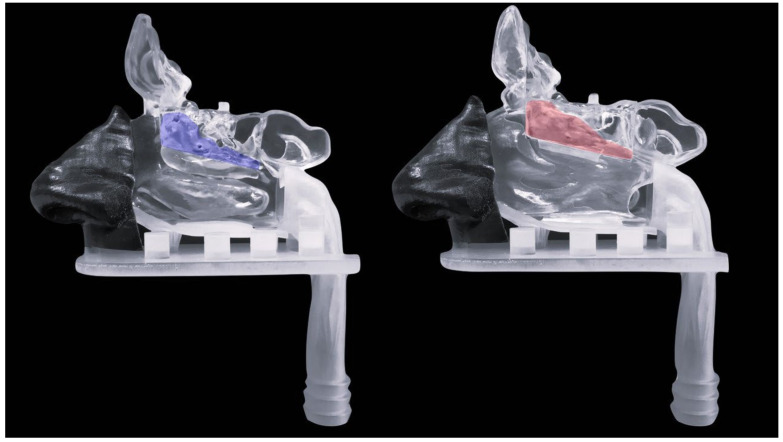
The olfactory region of the 3D-printed nasal cast: superior turbinate with a small portion of the middle turbinate (**left**, in blue) and corresponding segment of the nasal septum (**right**, in red).

The olfactory region corresponds to the upper turbinate with a small fragment of the middle turbinate and the corresponding part of the nasal septum (Figure 1). The respiratory region is presented by the rest of the turbinates and septum, lined with respiratory epithelium, and innervated by the trigeminal nerve [32]. 

The olfactory deposition pattern in the 3D-printed nasal cast used in this study was monitored for all DoE samples differing in formulation and administration parameters, and the results ranged from 1.9 ± 2.1 to 71.8 ± 0.8% (Table 2). Regression modelling produced a model that showed a good fit (R-squared 0.89, RMSE 8.83, Press R-squared 0.59 and Press RMSE 13.25), represented in the following equation:

OD (%) = 24.46 − 4.51 × *c*_DH_ − 5.74 × IFR* + 18.31 × AAH* + 4.48 × *c*_DH_ × IFR − 4.38 × *c*_DH_ × AAH − 4.63 × IFR × AAH (4)

The formulation parameters showed no significant influence on the deposition in the olfactory region. However, the equation above indicates that both administration parameters (AAH and IFR) have a significant influence on the olfactory deposition. The increase in the angle of administration from the horizontal plane (from 45° to 75°) increased the fraction of the drug deposited in the olfactory region. For liquid systems, high angles of administration have the potential to pass the nasal valve and aim for the olfactory region [56]. Indeed, in our study, the highest olfactory deposition was achieved at the highest angle of administration (at 75°), which is equal to 71.8 ± 0.8% of the administered dose, proving the assumption that the angle of administration is the critical factor for targeted olfactory deposition of nasal liquid sprays. 

The inspiratory flow rate was shown to decrease the olfactory deposition (Equation (4)). This could be due to the pattern of airflow during low to moderate breathing conditions. According to Tian et al. [85], during the moderate breathing flow, most air flows through the turbinate region (middle and inferior meatus), while airflow in the olfactory region stays very low to unchanged. It can be assumed that breathing and airflow steer the formulation from its original direction to the turbinate region and, as a result, a smaller fraction of the drug is deposited in the olfactory region. From the patient’s perspective, a ‘breath hold’ condition is preferred during the administration: less coordination between breathing and actuation leads to lower variability, and thus patient adherence to the therapy is facilitated [12,32]. 

For nasally administered neurological drugs, 0.01–1% of the orally applied dose needs to be delivered by nose-to-brain direct pathways to achieve its therapeutic potential [47]. In this study, the highest olfactory deposition attained by single-dose actuation represents 0.4% or 0.2% of oral daily dose (5 mg/10 mg) prescribed for the treatment of Alzheimer’s patients [86]. It is evident that the drug concentration set by the experimental design, coupled with an appropriate mode of administration, resulted in effective drug dosing. In addition, by nasal administration, the daily dose of donepezil can be reduced, which leads to reduced systemic bioavailability, with the potential to minimise side effects. 

##### Deposition in the Turbinate Region

The fraction of the deposited dose in the turbinate region for all DoE samples ranged between 10.5 ± 0.8 and 65.1 ± 3.3% (Table 2). The model retrieved from the regression modelling showed a good fit (R-squared 0.86, RMSE 9.41, Press R-squared 0.39 and Press RMSE 13.49) and is presented in the following equation:

TD (%) = 52.41 + 0.14 × *c*_DH_ + 2.38 × *c*_C_ + 5.24 × IFR − 11.42 × AAH* + 8.81 × *c*_DH_ × *c*_C_ *− 6.77 × *c*_DH_ × IFR* − 11.33 × IFR^2^ + 5.18 × IFR × AAH (5)

The administration angle measured from the horizontal plane had a significant impact on the turbinate deposition. It can be observed that the decrease in AAH led to an increase in the turbinate deposition. This effect has already been described in the literature [10,11,87], and it is explained by the fact that low angles, coupled with appropriate formulation parameters, have a great potential for delivering the formulation to the turbinate region, surpassing the nasal valve.

The inspiratory flow rate exhibited a quadratic effect on the turbinate deposition. A flow rate of 15 L min^−1^ showed a positive influence, which can be explained by the rise in air flow through the turbinate region [85]. The spray is carried by the airflow and the deposition is the result of convection. However, at 30 L min^−1^ airflow, a decrease in turbinate deposition was evident. At high airflows, liquid escapes the streamline and it is deposited by impaction [57]. The observed interaction between the DH concentration and the inspiratory flow rate indicates the combined impact of the formulation and administration parameters on spray aerodynamic properties.

The interaction between the formulation parameters (donepezil and chitosan concentrations) showed a positive effect on the turbinate deposition, which can be related the observed positive effect on solution viscosity and DSD. These characteristics, coupled with appropriate administration parameters, provided a greater potential for deposition beyond the nasal valve [70]. 

In conclusion, by using QbD methodology, we paved the way for the targeted drug delivery, resulting in an astoundingly high-dose fraction deposited in the olfactory region. So far, to the best of our knowledge, no studies have reported a fractional deposition pattern this high. The precise selection of components and their concentrations, followed by carefully selected administration parameters, resulted in an optimal DH nose-to-brain delivery. DH incorporated into a developed in situ gelling C-BGP platform showed great potential to achieve its therapeutic effect by a simple mode of nasal administration.

### 3.3. Selection of the Leading DH-C-BGP In Situ Gelling Formulation 

The design of the experiments, combining formulation and administration parameters, was used to identify the optimal DH-C-BGP in situ gelling formulation for nasal DH delivery. Guided by QbD principles and based on the identified quality target product profile (QTPP—development of thermosensitive DH-C-BGP platform for safe and efficient nose-to-brain delivery) during the formulation development, we assessed: the quality target product profile, critical quality attributes (CQA—spray characteristics: zero-shear viscosity, DSD, and plume geometry), thermogelling properties (gelation time and temperature), and nasal deposition profile and critical material attributes (CMA—concentration and type of compounds) [46].

The applied approach enabled the identification of the leading formulation, consisting of 0.3 mg mL^−1^ DH, 9.23 mg mL^−1^ C, and 188 mg mL^−1^ BGP (denoted as LF). The LF exhibited immediate gelling at the temperature of the nasal mucosa, and combined with a 75° administration angle measured from the horizontal plane at breath hold, resulted in the highest olfactory deposition (71.8 ± 0.8% of the applied dose). The selected formulation was characterised by a zero-shear viscosity of 206.37 ± 1.46 mPa s, a narrow plume angle (15.5 ± 0.4°), and an appropriate range of droplet sizes; all characteristics that are preferred for targeted olfactory deposition. The LF was further subjected to thorough biopharmaceutical characterisation to account for its performance in contact with the nasal mucosa.

### 3.4. In-Depth Characterisation of the Leading DH-C-BGP Formulation (LF)

#### 3.4.1. DH In Vitro Release Profile

**Figure 2:**
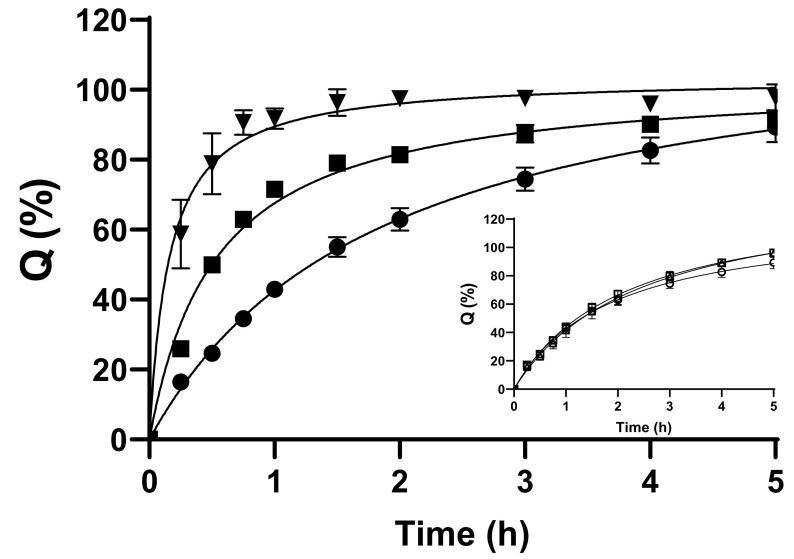
In vitro release profiles from the leading DH-C-BGP formulation (LF—circle) and the corresponding DH-C-BGP control formulation (CF—square) compared with the dissolution of the DH solution (reverse triangle). Graph insert: in vitro release profile of the LF immediately after preparation (empty circle), upon 30 days of storage (empty triangle), and upon 90 days of storage (empty square). Data are expressed as the mean ± SD, n = 3.

In vitro release studies were performed under sink conditions using an automated Franz diffusion system. The DH release profile from the LF was compared with two control samples: (i) the DH-C-BGP in situ gelling system differing from the LF in C concentration (control formulation—CF; chitosan concentrations in the leading and control samples were 9.23 and 6.15 mg mL^−1^, respectively) and (ii) the corresponding aqueous DH solution. SNF was used as a physiologically relevant acceptor medium. In order to stimulate the conditions at the nasal mucosa, the membranes placed between the donor and acceptor compartments were soaked in SNF for 15 min prior to the experiment, at 34 °C [88]. In vitro release profiles of the tested samples are presented in Figure 2. 

The drug release behaviour was studied over a 5 h period. It was observed that the DH release rate decreased in the following order: DH solution > CF > LF. The dissimilarity of all compared DH release profiles was confirmed using an *f*_2_ similarity criteria (*f*_2_ = 36.3 for the LF vs. the CF, *f*_2_ = 21.4 for the LF vs. the DH solution, and *f*_2_ = 35.4 for the CF vs. the DH solution).

Both the LF and the CF formed a gel at 34 °C. During the formation of the gel, its structure and viscosity determine the drug diffusion rate through the gel matrix and its release from the formulation [88,89]. Indeed, both thermosensitive formulations showed prolonged release (32.5 ± 4.0% and 62.9 ± 1.0% in 45 min for the LF and the CF, respectively) in comparison with the DH solution (90.6 ± 3.5% of the drug released in 45 min).

Due to different C concentrations and, consequently, different gelling times and viscosities, distinct release profiles for the LF and the CF were observed. The lower initial burst of DH from the LF can be explained by the fact that it exhibited instant gelation at 34 °C (i.e., the drug is entrapped in the gel from the beginning of the experiment), while the CF turned to gel after 1.0–1.3 min of exposure to 34 °C (Table 2). The overall slower drug release profile observed for the LF can be ascribed to higher viscosity and diffusion resistance of the gel prepared at higher chitosan concentrations [6,43,90]. The resultant sustained and efficient drug release, when coupled with prolonged residence time at the deposition site, bears the potential to increase drug absorption and bioavailability at the action site.

#### 3.4.2. Mucoadhesion 

Increasing nasal residence time is a crucial task for an efficient nasal drug delivery. It can be achieved by the sol–gel transition of the formulation in contact with the nasal mucosa and is also promoted by the use of mucoadhesive agents. These agents interact with mucins in the mucus layer, decreasing mucociliary clearance and improving formulation mucosal affinity [91].

**Figure 3:**
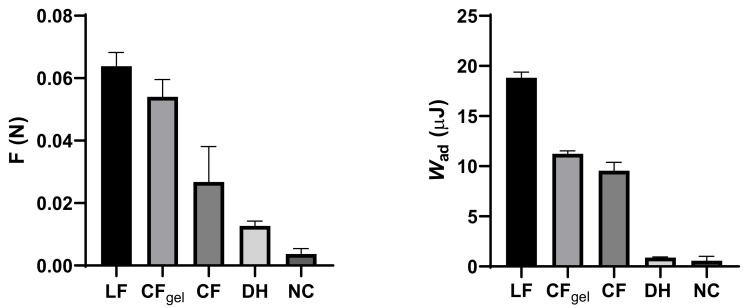
Maximum detachment force (*F*; **left**) and work of adhesion (*W*_ad_; **right**) of the leading DH-C-BGP formulation (LF), the control DH-C-BGP formulation (CF), the control DH-C-BGP formulation after gelation (CF_gel_), the DH aqueous solution (DH), and filter paper as the negative control (NC). Data are expressed as the mean ± SD, n = 3.

A mucoadhesion study of the LF was carried out using nasal porcine mucosa, on account of its physiological and histological similarity to human mucosa [92]. The following control samples were employed: (i) control formulation—CF (gelation time at 34 °C of the CF is 1.0–1.3 min (Table 2); and (ii) corresponding aqueous DH solution (DH). Mucoadhesion of the CF was determined: (i) under the described test conditions applied for all samples (sol–gel transition of the CF appeared in the last third of the testing time), and employing longer thermostating (2 min) to ensure gelation before the start of the test (CF_gel_). Filter paper served as a negative control (NC). The obtained results are presented in Figure 3.

The LF showed prominent mucoadhesive properties, with a five-fold higher maximum detachment force and a 20-fold higher work of adhesion in relation to the drug solution. The LF presented a 2.4-fold higher maximum detachment force and 2.0-fold higher work of adhesion in relation to the CF formulation. The obtained results indicate a largely improved nasal retention potential of the LF in relation to the DH simple solution and an impact of gelation time and chitosan concentration on its mucoadhesive performance. To differentiate between these two effects, CF_gel_ was also analysed for its mucoadhesive properties. Comparing the results for the CF and the CF_gel_, it was observed that the sol–gel transition contributed significantly to the detachment force (*p* = 0.019). At the same time, the comparison of the leading and control gelled formulations (LF and CF_gel_) suggested a significant influence of chitosan concentration on the work of adhesion (*p* < 0.001).

The chitosan’s mechanism of mucoadhesion is a result of alectrostatic interaction between positively charged amino groups of chitosan and negatively charged mucins of the nasal mucosa [93]. It is important to note that the pH of the leading formulation is about neutral (pH = 7.02). At this pH, amino groups of chitosan are not fully protonated and the percentage of chitosan ionisation at the pH of the formulation can be calculated according to the Henderson–Hasselbalch equation [94]. Considering the pKa of chitosan (pKa = 6.5 [95]), the percentage of ionisation of chitosan in the DH-C-BGP is 23.1%. Nonetheless, a moderate electrostatic interaction might contribute to a better mucosal tolerability of chitosan [42,96]. 

Chitosan also interacts with mucin through other attractive forces—hydrogen bonding and hydrophobic interaction [97]. Higher values of detachment force and work of adhesion for formulations with higher chitosan concentrations can be explained by all the above-mentioned mechanisms of chitosan mucoadhesivness.

#### 3.4.3. In Vitro Biocompatibility 

Excipients used in the formulation of DH thermosensitive gelling systems are known to be safe—applied individually or in combination [27,29,43]. As stated earlier, chitosan is a non-toxic and biocompatible polymer [98] and BGP is a substance naturally present in the human body that is also approved by the Food and Drug Administration (FDA) as a parenteral phosphate supplement [51]. 

The human airway epithelial Calu-3 cell line was used to evaluate the biocompatibility of the LF. The LF was diluted with HBSS/HEPES (pH = 7.0) in a 1:1 volume ratio, resulting in DH, C, and BGP concentrations of 0.15 mg mL^−1^, 4.62 mg mL^−1^, and 94.00 mg mL^−1^, respectively. The DH solution (0.05 and 0.25 mg mL^−1^) and BGP solution (94.00 mg mL^−1^) served as controls. Pure HBSS/HEPES (pH = 7.0) served as a negative control. The cells’ incubation temperature (37 °C) induced the gel formation of the leading formulation. No cytotoxic effect was observed in any of the tested formulations. The viability of the Calu-3 cells was above 80% in relation to the control (86.0 ± 1.4% for the LF; 86.4 ± 2.5% and 99.1 ± 9.9% for 0.05 mg mL^−1^ and 0.25 mg mL^−1^ for the DH solutions, respectively; and 95.7 ± 9.7% for the BGP solution). At the pH of the leading formulation, chitosan is not fully protonated, as discussed in Section 3.4.2. Positive amino groups of chitosan interact with negatively charged cell membranes and this interaction can lead to a reduction in cell viability. However, a lower extent of chitosan protonation at a higher pH, or shielding the positive charges on the chitosan molecule, can result in a decreased chitosan cytotoxicity [96,99]. The obtained results showed appropriate biocompatibility of the LF at the Calu-3 cell model. The selected compound concentrations are recognised to be optimal for further permeability studies.

#### 3.4.4. In Vitro DH Permeability 

Permeability studies were performed using Calu-3 cells grown at an air–liquid interface. Calu-3 cells form monolayers that adequately simulate the nasal epithelial barrier considering its ultrastructure, mucus production, and barrier properties [100], while providing benefits of immortalised cell lines including high reproducibility and genetic homogeneity [101]. Recently, the Calu-3 cell model was successfully used by our group to screen chitosan-based powder formulations intended for DH nose-to-brain delivery [12].

In this work, the permeability study was designed to screen the permeation enhancing potential of the LF compared with the DH solution and to differentiate between the effect of chitosan and hyperosmotic conditions on monolayer integrity and the DH permeation profile. Thus, the following samples were tested on Calu-3 cell monolayers: (i) the LF mixed with HBSS/HEPES (pH = 7.0) in a ratio of 1:1, *v*/*v* (final DH concentration 0.15 mg mL^−1^; osmolality 750 ± 3 mOsm/kg), and (ii) the DH solution in hyperosmolar HBSS/HEPES (pH = 7.0) (DH concentration 0.15 mg mL^−1^; osmolality 745 ± 9 mOsm/kg) and DH solution in HBSS/HEPES (pH = 7.0) (DH concentration 0.25 mg mL^−1^; osmolality 340 ± 1 mOsm/kg). The permeability study was performed under sink conditions, and assuming predominant passive paracellular transport of the hydrophilic drug, no influence of DH concentration in the donor/receiver compartment on DH permeation could be expected [102,103]. Transepithelial electric resistance (TEER) was monitored over the experiment and up to 24 h upon the start of the experiment, as an indicator of the monolayer integrity.

The results of permeability studies of DH from the tested samples across the Calu-3 cell monolayer are given in Table 4. The *P*_app_ value of the DH from the solution in HBSS/HEPES (pH 7.0) (DH concentration 0.25 mg mL^−1^; *P*_app_ = 3.38 × 10^−5^ cm s^−1^) evaluated in this study is in line with the previously obtained *P*_app_ value for the DH solution in HBSS-Ca^2+^/HEPES (pH = 6.0) (DH concentration 0.008 mg mL^−1^; osmolality 321 ± 1 mOsm/kg; *P*_app_ = 3.59 × 10^−5^ cm s^−1^) using the same cell model [12].

**Figure 4:**
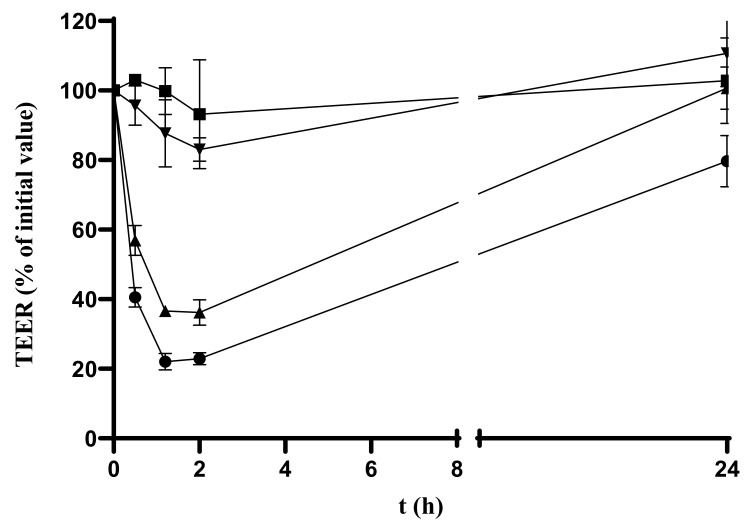
Trans-epithelial electric resistance (TEER) values of the Calu-3 cell monolayer during the study of DH permeability from the LF (circle), the DH solution in hyperosmolar HBSS/HEPES (pH = 7.0) (triangle), the DH solution in HBSS/HEPES (pH = 7.0) (square), and HBSS/HEPES (pH = 7.0) (reverse triangle). Data are expressed as the mean ± SD, n = 3.

The *P*_app_ value of the DH from the LF was 1.47-fold higher than that of the DH solution, confirming the formulation’s permeation-enhancing effect. Considering the prolonged DH release observed in in vitro release studies, it may be concluded that the permeation-enhancing effect was even higher than described with the calculated attenuation factor. Namely, in the case of the LF, only the released fraction of the drug was available for permeation at each time point, while in the case of drug solution, the total dose of dissolved drug is instantly available for permeating the cell monolayer [12,104]. The permeation-enhancing effect of the LF can be explained by the observed decrease in the TEER value of the cell monolayer (Figure 4), indicating the loosening of the barrier properties and favouring paracellular transport of a hydrophilic drug. 

The reversible nature of the LF effect on the barrier integrity was confirmed by the TEER increase to 80 ± 7% of the initial value 22 h after the formulation was washed off the cell monolayer. The observed TEER decrease was ascribed to the reversible opening of the tight junctions resulting from a combined effect of the chitosan and the hyperosmolality of the formulation. Such a conclusion was supported by a more pronounced TEER decrease observed for the leading hyperosmolar chitosan-based formulation than for the chitosan-free hyperosmolar DH solution (Figure 4). Interestingly, the hyperosmolar DH solution showed no permeation-enhancing effect compared with the isoosmolar DH solution (attenuation factor = 0.98), stressing the role of chitosan in promoting DH permeation across the cell monolayer barrier.

#### 3.4.5. Slug Mucosal Irritation Assay 

Due to the intense contact of the applied nasal formulation and the sensitive nasal mucosa, certain nasal discomfort, described as stinging, itching, and burning (SIB) sensations, may occur. These adverse effects affect patient compliance, and consequently the outcome of the treatment [105]. For this reason, it is very important to assess the formulation’s potential for irritation in the early stage of formulation development. Adriaens and Remon [106] developed a simple and inexpensive method to predict the formulation’s potential irritancy on the mucosal surfaces. The slug mucosal irritation (SMI) assay uses slugs of species *Arion lusitanicus* as a test organism to assess the irritation potency. Slugs are placed on a tested substance. If the formulation causes irritation, the slug produces mucus as a protective mechanism [107]. Based on the total amount of the produced mucus, and in relation to the positive and negative controls, it is possible to assess the irritation potential of the formulation and predict the discomfort the formulation may cause to the patient [37]. 

We performed the SMI assay as described earlier [37]. The mucus production after exposing the slug to the LF was compared with the results of the SMI assay performed on the positive control (maximum irritation) and the negative control (no irritation). The results of the performed SMI assay are presented in Table 5.

The mucus production for the LF was 2.7 times lower compared with the BAC 1% (*w*/*v*), which is used as a marker for severe discomfort [108]. The observed LF-induced mucus production was comparable to a previously reported result obtained for mannitol (sieved fraction 32–90 µm; 6.30 ± 0.61%), employing the same SMI assay. The observed mucus production was ascribed to a size-related dissolution rate and osmotic effect [37]. 

The tolerability and formulation potential to cause irritation can be ascribed to formulation pH, constituents, and osmolality [105]. Considering the relatively high mucus production previously observed for carboxymethyl chitosan powder [37], it may be assumed that the neutral pH of the LF contributed to its tolerability, as a neutral pH renders the chitosan charge density to a moderately low value. 

The hypertonic nature of thermosensitive chitosan/polyol-phosphate in situ gelling systems has already been reported [43,51,109]. Lenoir et al. tested a hypertonic NaCl solution (2.6%, *w*/*v*), characterised by similar osmolality to the LF, employing a human nose irritation test [105]. After 5 min of the nasal exposure, 54% of the participants did not feel discomfort, while 41.5% experienced mild discomfort. Ten minutes after the exposure, 79% of the participants did not feel discomfort anymore. 

It can be concluded that the LF in the SMI assay showed an acceptable irritability profile, demonstrating its potential for safe nasal delivery.

#### 3.4.6. Stability Profile

The physicochemical stability of the LF was monitored over a period of 3 months. The formulation was stored in an impermeable container at 5 ± 3 °C and inspected for drug concentration, rheological and gelling properties (zero-shear viscosity, and gelation temperature and time), spray characteristics, and drug release profile. Stability studies revealed no significant change in any of the tested characteristics, confirming the suitability of the formulation stability profile (Table 6). The *f*_2_ criteria for similarity estimation revealed no significant difference between release profiles of the LF determined after 0, 30, and 90 days of storage at described conditions (Table 6; Figure 2, insert).

### 3.5. Discussing the Potential of the Developed DH Liquid Formulation in Comparison with the DH Powder Formulation 

In this study, a thermoresponsive chitosan-based in situ gelling system was proven to be a promising platform for an efficient nasal DH delivery. Recently, our research group developed a comparable nasal spray-dried DH formulation, embedding the well-known advantages of a dry nasal form [12]. The applied complementary approach, including the development of both powder and liquid DH delivery platforms, is beneficial considering the patient perspective, as patients still prefer liquid formulations over nasal powders [110,111]. This aspect is particularly important in the targeted patient population since the adherence of AD patients is linked to the tolerability of drug therapy [112].

The comparison between powder and liquid formulations, based on their advantages and disadvantages, is presented in the literature [2,5]. However, to the best of our knowledge, there are no reports in which the comparability between powder and liquid platforms of the same active substance has been discussed, particularly in terms of nasal deposition profiles.

Both technological platforms, apart from being of simple production and acceptable stability profile, allowed for the optimisation of physicochemical, biopharmaceutical, and nasal deposition properties, which are crucial for an efficient DH nose-to-brain delivery. Indeed, a high olfactory deposition was obtained with both liquid (71.8% of the applied dose) and powder (65.5% of the applied dose) formulations [12], despite distinct aerodynamic properties of dispersed droplets and dried particles. The direct comparison of deposition profiles is feasible since the same 3D-printed model of the nasal cavity was used for the evaluation of both types of formulations. The high olfactory deposition was achieved by a fine-tuning of administration parameters in relation to formulation-specific properties. Generally, different administration angles from the horizontal plane were found to favour an olfactory deposition of aerosolised droplets and dry particles, while inspiratory airflow reduced the olfactory deposition of both formulations. Complex regression models for olfactory deposition efficiency, coupling formulation properties, and mode of administration stress the need for deposition considerations in the early phase of the formulation development. 

Finally, the comparison of distinct liquid and powder DH formulations using an animal model is needed to rate their in vivo performance in terms of potentiating DH brain bioavailability.

## 4. Conclusions

A thermogelling chitosan-based donepezil formulation was prepared by a fast, single-step method applicable in industrial settings. The manufacturing process enabled the fine-tuning of the formulation parameters that resulted in a final product of desirable characteristics. Droplet size, rheology, and sprayability were optimised features of the nasal product that, coupled with appropriate administration parameters, resulted in an efficient olfactory deposition (71.8% of the applied dose), which is crucial for the therapeutic outcome of nasally applied donepezil. The optimised formulation proved to be stable at the observed period of time and showed biopharmaceutical properties, suggesting the potential for safe nasal administration, prolonged retention at the nasal mucosa, sustained DH release, and increased permeability across the epithelial barrier. The obtained results indicate the formulation potential to promote DH brain bioavailability and support the continuation of studies aimed at gaining in vivo proof-of-concept and comparison of distinct liquid and powder DH formulations.

## Figures and Tables

**Table 1:** Settings of the parameters considered in the experimental design.

Parameter	High (+1)	Medium (0)	Low (−1)
Donepezil hydrochloride (DH) concentration (mg mL^−1^)	0.50	0.40	0.30
Chitosan (C) concentration (mg mL^−1^)	9.23	7.69	6.15
Angle of administration from the horizontal plane, AAH (°)	75	60	45
Inspiratory flow rate, IFR (L min^−1^)	30	15	0

**Table 4:** Osmolality and DH apparent permeability coefficients (*P*_app_) across the Calu-3 monolayer of the LF, the DH solution in hyperosmolar HBSS/HEPES (pH = 7.0), and the DH solution in HBSS/HEPES (pH = 7.0).

Sample	Osmolality (mOsm kg^−1^)	*P*_app_ (10^−5^ cm s^−1^)	Attenuation Factor
Leading DH-C-BGP formulation (LF)	750 ± 3	4.96 ± 0.84	1.47
DH solution in hyperosmolar HBSS/HEPES (pH = 7.0)	745 ± 9	3.31 ± 2.60	0.98
DH solution in HBSS/HEPES (pH = 7.0)	340 ± 1	3.38 ± 3.23	-
HBSS/HEPES (pH = 7.0)	312 ± 2	-	-

The attenuation factor was calculated as the ratio between the *P*_app_ value of the LF or DH solution in hyperosmolar HBSS/HEPES (pH = 7.0) and the *P*_app_ value of the DH solution in HBSS/HEPES (pH = 7.0). Values are the mean ± SD, n = 3.

**Table 5:** Total mucus production from the three contact periods in the SMI assay expressed as a percentage of the initial body weight of the slugs. The results are expressed as the mean ± SD, n = 3.

Sample	Total Mucus Production (%)
Leading DH-C-BGP formulation (LF)	6.64 ± 1.04
PBS—negative control	0.48 ± 1.50
BAC 1% (*w*/*v*)—positive control	17.64 ± 4.33

**Table 6:** Three-month stability data for the leading DH-C-BGP in situ gelling formulation.

Inspected Property	Immediately after Preparation	After 30 Days	After 90 Days
DC (mg mL^−1^)	0.30 ± 0.00	0.30 ± 0.00	0.30 ± 0.01
*η*_0_ (mPa s)	206.37 ± 1.46	192.28 ± 11.57	211.02 ± 6.53
*T*_GEL_ (°)	33.7 ± 0.1	33.9 ± 0.3	34.9 ± 0.1
*t_GEL_* (min)	0.0 ± 0.0	0.0 ± 0.0	0.0 ± 0.0
*D*_v_10 (μm)	69.8 ± 3.7	74.6 ± 6.0	70.2 ± 1.4
*D*_v_50 (μm)	161.1 ± 5.2	167.2 ± 4.5	166.5 ± 2.6
*D*_v_90 (μm)	309.7 ± 10.9	313.1 ± 3.9	317.4 ± 7.7
Span	1.5 ± 0.0	1.4 ± 0.1	1.5 ± 0.0
SCA (°)	15.5 ± 0.4	15.6 ± 0.2	15.8 ± 0.1
*f*_2_	-	72.7	70.2

DC = drug concentration; *η*_0_ = zero-shear viscosity; *T*_GEL_ = gelation temperature; *t_GEL_* = gelation time; *D*_v_10; *D*_v_50 and *D*_v_90 = droplet size distribution; SCA = spray cone angle; and *f*_2_ = similarity factor of in vitro release profiles after 30 and 90 days compared with the release profile immediately after preparation.

## Data Availability

Not applicable.

## Supplementary Materials

The following supporting information can be downloaded at: https://www.mdpi.com/article/10.3390/pharmaceutics15061660/s1.
