# Supplementary material for: Chitosan-Based Thermogelling System for Nose-to-Brain Donepezil Delivery: Optimising Formulation Properties and Nasal Deposition Profile
Source: Pharmaceutics. 2023 Jun 5;15(6):1660. doi: 10.3390/pharmaceutics15061660 (PMC10302257; doi:10.3390/pharmaceutics15061660)
Supplement: Supplementary file 1 [file pharmaceutics-15-01660-s001.zip › Supplementary Figures.pdf]

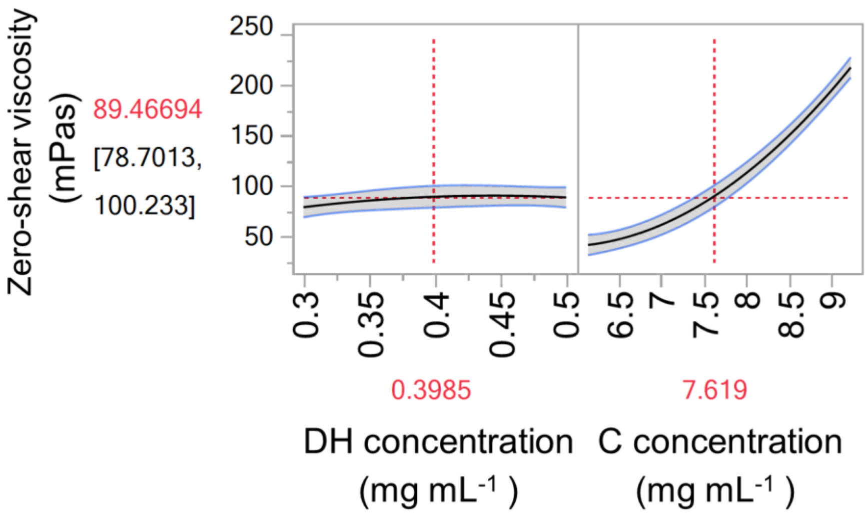

Figure S1. Prediction of zero-shear viscosity in relation to donepezil (DH) and low molecular weight chitosan (C) concentration. Values in the brackets refer to 95% confidence interval.

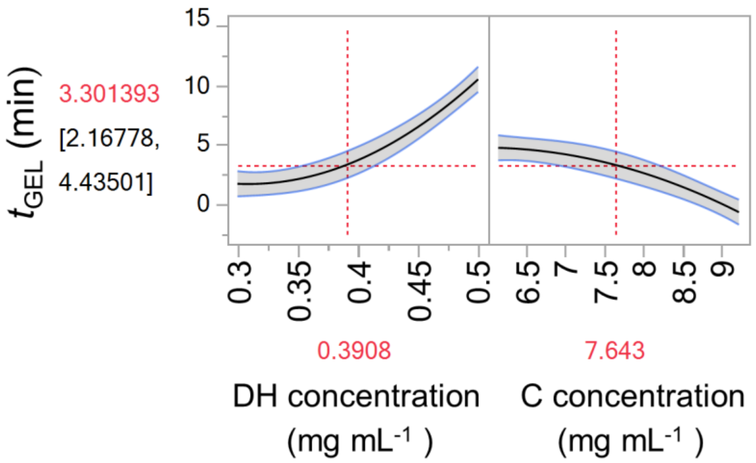

Figure S2. Prediction of gelation time (t_{gel}) in relation to donepezil (DH) and low molecular weight chitosan (C) concentration. Values in the brackets refer to 95% confidence interval.

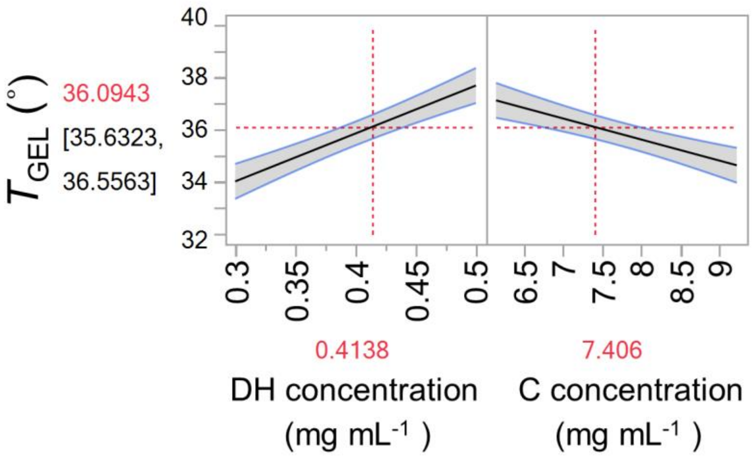

Figure S3. Prediction of gelation temperature (T_{gel}) in relation to donepezil (DH) and low molecular weight chitosan (C) concentration. Values in the brackets refer to 95% confidence interval.

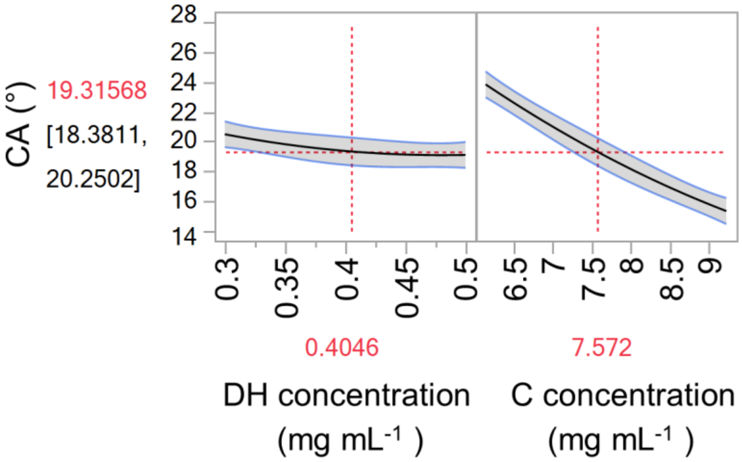

Figure S4. Prediction of spray cone angle (CA) in relation to donepezil (DH) and low molecular weight chitosan (C) concentration. Values in the brackets refer to 95% confidence interval.

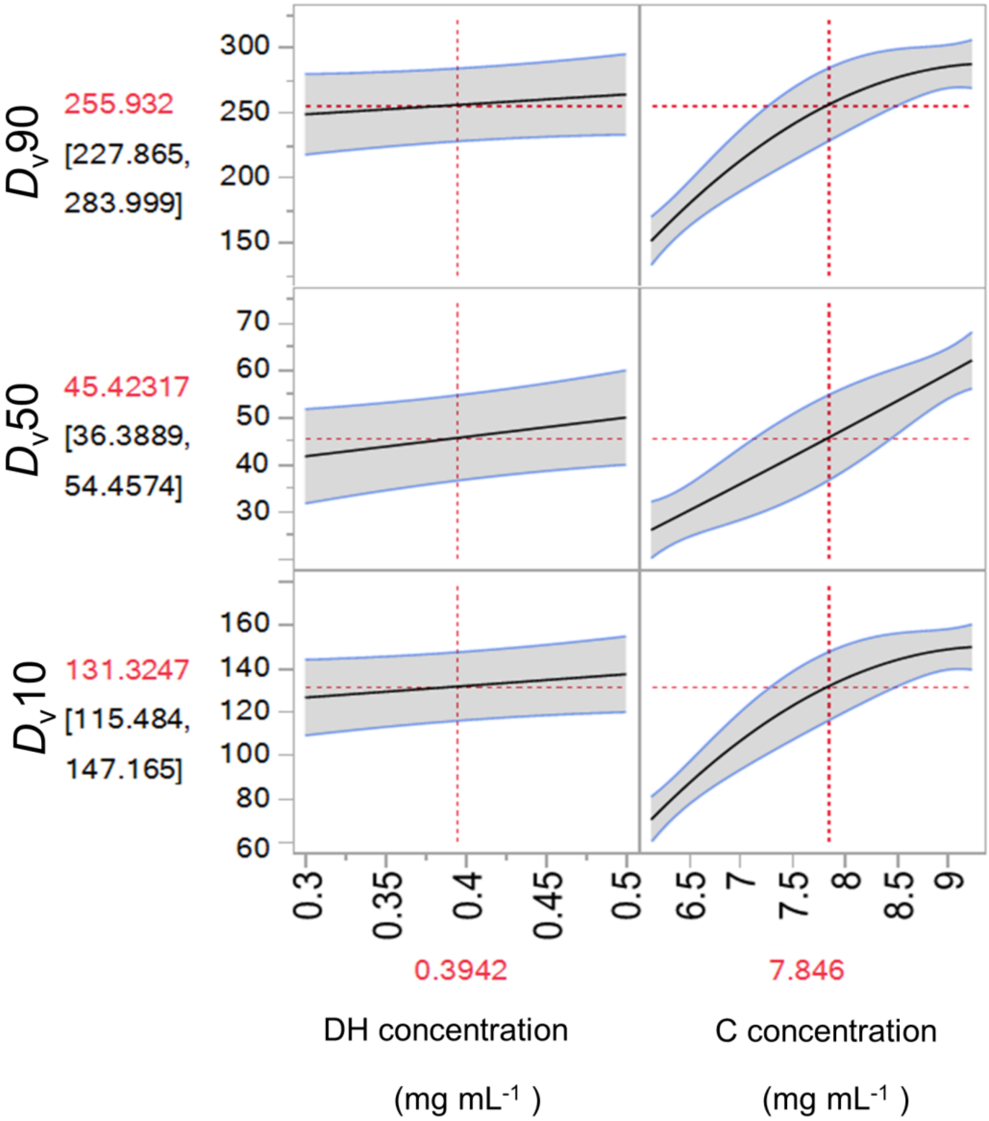

Figure S5. Prediction of droplet size distribution (D_v10, D_v50 and D_v90) in relation to donepezil (DH) and low molecular weight chitosan (C) concentration. Values in the brackets refer to 95% confidence interval.

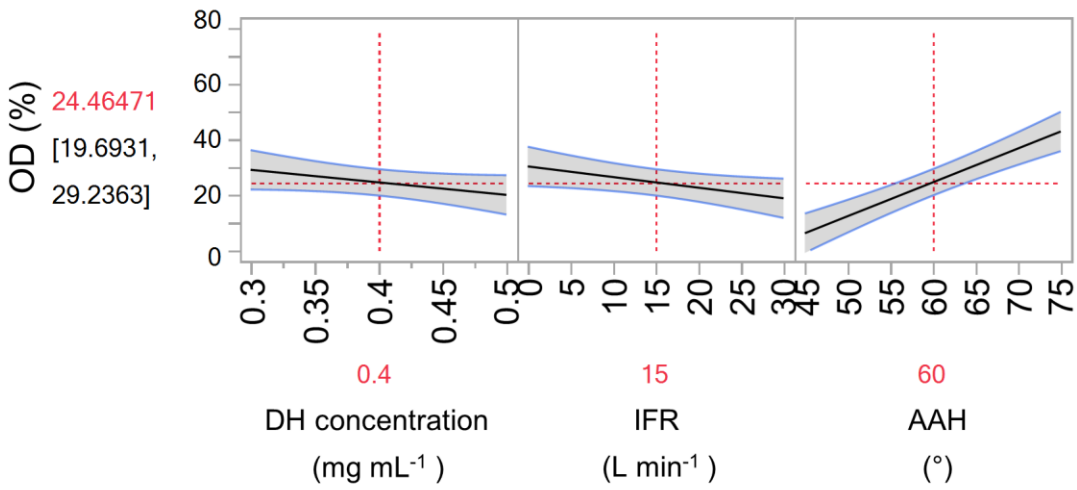

Figure S6. Prediction of olfactory deposition (OD) in relation to donepezil (DH) concentration, inspiratory flow rate (IFR) and angle of administration from the horizontal angle (AAH). Values in the brackets refer to 95% confidence interval.

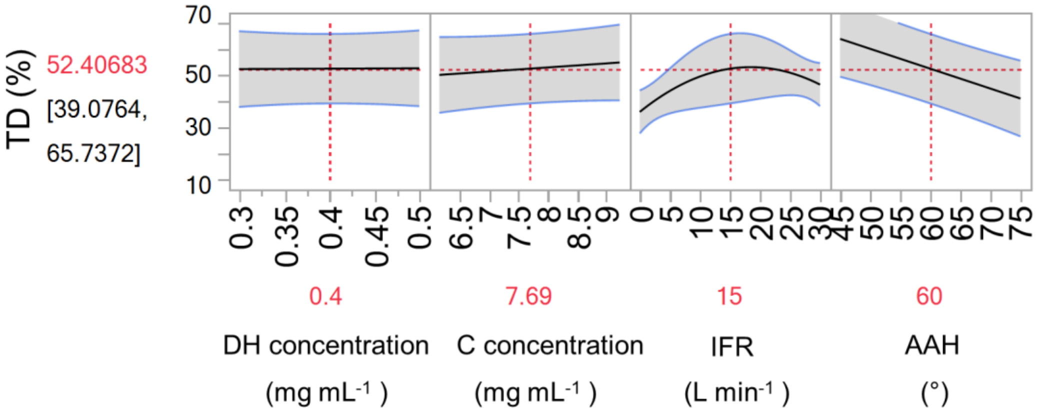

Figure S7. Prediction of turbinate deposition (TD) in relation to donepezil (DH) and low molecular weight chitosan (C) concentration, inspiratory flow rate (IFR) and angle of administration from the horizontal angle (AAH). Values in the brackets refer to 95% confidence interval.
