# Supplementary material for: Chitosan-Based Thermogelling System for Nose-to-Brain Donepezil Delivery: Optimising Formulation Properties and Nasal Deposition Profile
Source: Pharmaceutics. 2023 Jun 5;15(6):1660. doi: 10.3390/pharmaceutics15061660 (PMC10302257; doi:10.3390/pharmaceutics15061660)
Supplement: Supplementary file 1 [file pharmaceutics-15-01660-s001.zip › Table S1.pdf]

Table S1. Validation parameters of HPLC method employed for quantitative determination of DH.

Range of linearity					
1 – 100 μg mL^{-1}					
Linearity					
regression equation			correlation coefficient		
y = 31.918x – 30.131			0.9996		
Precision data					
repeatability (RSD %)			intermediate precision (RSD %)		
low<br>10 μg mL^{-1}	medium<br>50 μg mL^{-1}	high<br>90 μg mL^{-1}	low<br>10 μg mL^{-1}	medium<br>50 μg mL^{-1}	high<br>90 μg mL^{-1}
0.1	0.1	0.0	2.6	1.0	4.7
Accuracy Data<br>accuracy (recovery, mean (%) ± SD, n = 3)					
low<br>(10 μg mL^{-1})		medium<br>(50 μg mL^{-1})		high<br>(90 μg mL^{-1})	
97.9 ± 0.1		98.1 ± 0.1		98.8 ± 0.0	
Sensitivity					
LOD (μg mL^{-1})			LOQ (μg mL^{-1})		
0.95			2.88		

* x - concentration (μg mL^{-1}); y –Area under Curve (mAU); All measurements are performed in triplicate.
