# Supplementary material for: Chitosan-Based Thermogelling System for Nose-to-Brain Donepezil Delivery: Optimising Formulation Properties and Nasal Deposition Profile
Source: Pharmaceutics. 2023 Jun 5;15(6):1660. doi: 10.3390/pharmaceutics15061660 (PMC10302257; doi:10.3390/pharmaceutics15061660)
Supplement: Supplementary file 1 [file pharmaceutics-15-01660-s001.zip › Table S2.pdf]

Table S2. Visual inspection of the preliminary samples prepared – selection of the chitosan type.

Chitosan concentration 6.15 mg mL^{-1}	BGP concentration 188.00 mg mL^{-1}			
	DH concentration (mg mL^{-1})			
**Type of chitosan**	**0.30**	**0.40**	**0.50**	**0.60**
**LOW molecular weight**	clear	clear	clear	precipitation
**MEDIUM molecular weight**	clear	precipitation	precipitation	precipitation
**HIGH molecular weight**	clear	precipitation	precipitation	precipitation
