# Supplementary material for: Chitosan-Based Thermogelling System for Nose-to-Brain Donepezil Delivery: Optimising Formulation Properties and Nasal Deposition Profile
Source: Pharmaceutics. 2023 Jun 5;15(6):1660. doi: 10.3390/pharmaceutics15061660 (PMC10302257; doi:10.3390/pharmaceutics15061660)
Supplement: Supplementary file 1 [file pharmaceutics-15-01660-s001.zip › Table S3.pdf]

Table S3. Gelation and spray properties of the preliminary samples – selection of chitosan type.

**Chitosan concentration 6.15 mg mL^{-1}**	**BGP concentration 188.00 mg mL^{-1} DH concentration 0.30 mg mL^{-1}**	
**Type of chitosan**	**Gelation at 34 °C**	**Sprayability**
**LOW molecular weight**	instant	adequate
**MEDIUM molecular weight**	1.33 min	poor
**HIGH molecular weight**	instant	poor
