# Supplementary material for: Chitosan-Based Thermogelling System for Nose-to-Brain Donepezil Delivery: Optimising Formulation Properties and Nasal Deposition Profile
Source: Pharmaceutics. 2023 Jun 5;15(6):1660. doi: 10.3390/pharmaceutics15061660 (PMC10302257; doi:10.3390/pharmaceutics15061660)
Supplement: Supplementary file 1 [file pharmaceutics-15-01660-s001.zip › Table S4.pdf]

Table S4. Optimisation of the BGP concentration in the preliminary samples.

**BGP concentration ($mg\ mL^{-1}$)**	**Low molecular weight chitosan concentration 6.15 $mg\ mL^{-1}$**
	**DH concentration 0.30 $mg\ mL^{-1}$**
	**Gelation properties**
**176.00**	thermogelling properties are not exhibited at the temperature range of the nasal cavity
**188.00**	appropriate thermogelling properties
**200.00**	gel properties exhibited at room temperature
