# Supplementary material for: Chitosan-Based Thermogelling System for Nose-to-Brain Donepezil Delivery: Optimising Formulation Properties and Nasal Deposition Profile
Source: Pharmaceutics. 2023 Jun 5;15(6):1660. doi: 10.3390/pharmaceutics15061660 (PMC10302257; doi:10.3390/pharmaceutics15061660)
Supplement: Supplementary file 1 [file pharmaceutics-15-01660-s001.zip › Table S5.pdf]

Table S5. Visual inspection of the preliminary samples – setting the DH and low molecular weight chitosan concentration for the design of experiments.

**LOW molecular weight chitosan concentration (mg mL^{-1})**	**BGP concentration 188.00 mg mL^{-1}**		
	**DH concentration (mg mL^{-1})**		
	**0.30**	**0.40**	**0.50**
**3.08**	clear	clear	clear
**6.15**	clear	clear	clear
**7.69**	clear	clear	clear
**9.23**	clear	clear	clear
**12.31**	clear	clear	clear
