# Supplementary material for: Chitosan-Based Thermogelling System for Nose-to-Brain Donepezil Delivery: Optimising Formulation Properties and Nasal Deposition Profile
Source: Pharmaceutics. 2023 Jun 5;15(6):1660. doi: 10.3390/pharmaceutics15061660 (PMC10302257; doi:10.3390/pharmaceutics15061660)
Supplement: Supplementary file 1 [file pharmaceutics-15-01660-s001.zip › Table S6.pdf]

Table S6. Gelation and spray properties of the preliminary samples – setting the DH and low molecular weight chitosan concentration for the design of experiments.

LOW molecular weight chitosan concentration (mg mL^{-1})	BGP concentration 188.00 mg mL^{-1}		
	DH concentration (mg mL^{-1})		
	0.30	0.40	0.50
	Gelation and spray properties		
3.08	adequate sprayability / thermogelling properties are not exhibited at the temperature range of the nasal cavity		
6.15	adequate sprayability / appropriate thermogelling properties		
7.69	adequate sprayability / appropriate thermogelling properties		
9.23	adequate sprayability / appropriate thermogelling properties		
12.31	poor sprayability / gel properties exhibited at room temperature		
